# Incidence and prevalence of dementia associated with transient ischaemic attack and stroke: analysis of the population-based Oxford Vascular Study

**DOI:** 10.1016/S1474-4422(18)30442-3

**Published:** 2019-03

**Authors:** Sarah T Pendlebury, Peter M Rothwell

**Affiliations:** a Centre for Prevention of Stroke and Dementia, Nuffield Department of Clinical Neurosciences, John Radcliffe Hospital and University of Oxford, Oxford, UK

## Abstract

**Background:**

Risk of dementia after stroke is a major concern for patients and carers. Reliable data for risk of dementia, particularly after transient ischaemic attack or minor stroke, are scarce. We studied the risks of, and risk factors for, dementia before and after transient ischaemic attack and stroke.

**Methods:**

The Oxford Vascular Study is a prospective incidence study of all vascular events in a population of 92 728 people residing in Oxfordshire, UK. Patients with transient ischaemic attack or stroke occurring between April 1, 2002, and March 31, 2012, were ascertained with multiple methods, including assessment in a dedicated daily emergency clinic and daily review of all hospital admissions. Pre-event and post-event (incident) dementia were diagnosed at initial assessment and during 5-years' follow-up on the basis of cognitive testing supplemented by data obtained from hand searches of all hospital and primary care records. We assessed the association between post-event dementia and stroke severity (as measured with the US National Institutes of Health Stroke Scale [NIHSS] score), location (ie, dysphasia), previous events, markers of susceptibility or reserve (age, low education, pre-morbid dependency, leucoaraiosis), baseline cognition, and vascular risk factors with Cox regression models adjusted for age, sex, and education. We compared incidence and prevalence of dementia in our population with published UK population age-matched and sex-matched rates.

**Findings:**

Among 2305 patients (mean age 74·4 years [SD 13·0]), 688 (30%) had transient ischaemic attacks and 1617 (70%) had strokes. Pre-event dementia was diagnosed in 225 patients; prevalence was highest in severe stroke (ie, NIHSS >10) and lowest in transient ischaemic attack. Of 2080 patients without pre-event dementia, 1982 (95%) were followed up to the end of study or death. Post-event dementia occurred in 432 of 2080 patients during 5 years of follow-up. The incidence of post-event dementia at 1 year was 34·4% (95% CI 29·7–41·5) in patients with severe stroke (NIHSS score >10), 8·2% (6·2–10·2) in those with minor stroke (NIHSS score <3), and 5·2% (3·4–7·0) in those with transient ischaemic attack. Compared with the UK age-matched and sex-matched population, the 1-year standardised morbidity ratio for the incidence of dementia was 47·3 (95% CI 35·9–61·2), 5·8 (4·4–7·5), and 3·5 (2·5–4·8), respectively. Consequently, prevalence of dementia in 1-year survivors was brought forward by approximately 25 years in those who had severe strokes, 4 years in those who had minor strokes, and 2 years in those who had transient ischaemic attacks. 5-year risk of dementia was associated with age, event severity, previous stroke, dysphasia, baseline cognition, low education, pre-morbid dependency, leucoaraiosis, and diabetes (p<0·0001 for all comparisons, except for previous stroke [p=0·006]).

**Interpretation:**

The incidence of dementia in patients who have had a transient ischaemic attack or stroke varies substantially depending on clinical characteristics including lesion burden and susceptibility factors. Incidence of dementia is nearly 50 times higher in the year after a major stroke compared with that in the general population, but excess risk is substantially lower after transient ischaemic attack and minor stroke.

**Funding:**

Wellcome Trust, Wolfson Foundation, British Heart Foundation, National Institute for Health Research, and the National Institute for Health Research Oxford Biomedical Research Centre.

## Introduction

Reliable estimates of the incidence of dementia after transient ischaemic attack and stroke are required to counsel patients and families and to inform prevention trials. However, most available data are from cross-sectional studies and hospital-based cohorts of major stroke, which are subject to selection biases and were done before the advent of robust secondary prevention.^1^, ^2^ Stroke has been estimated to bring forward the onset of dementia by about 10 years,^3^ but there are few data on the effect of event severity, particularly the risk after transient ischaemic attacks or minor stroke, which make up around 70% of all acute cerebrovascular events and often result in anxiety in patients and families about future risks.^4^ Anxiety could be compounded by the reportedly high rates of cognitive impairment after transient ischaemic attack,^5^, ^6^ and by public education suggesting high incidence of dementia.^7^ The frequency of progression of cognitive impairment to dementia after transient ischaemic attack and minor stroke is unclear, as is the extent to which the prevalence and incidence of dementia are higher among those who have had transient ischaemic attacks or minor strokes than those expected among the age-matched general population.

**Research in context**

**Evidence before this study**

We updated our previous systematic review of stroke-associated dementia (in which we searched Ovid and MEDLINE from Jan 1, 1950, to May 1, 2009, and Embase from Jan 1, 1980, to April 30, 2009), by searching these databases with the exploded medical subject headings [dementia] OR [vascular dementia] OR [multi-infarct dementia] AND [stroke] for articles published in English up to May 31, 2018. Pooled estimates showed that pre-stroke dementia was present in around 10% of patients before stroke, and that new dementia occurred in around 25% of people admitted to hospital with stroke in the first year after stroke. Hazard ratios for dementia among patients who have had strokes compared with the non-stroke population ranged from 2–8, with highest incidence of dementia in the immediate post-stroke period. However, most available data were for major stroke. Few population-based or longitudinal studies have been done overall, and few studies have been done since the advent of robust secondary prevention. Data were also scarce for dementia after transient ischaemic attack and minor stroke, which account for 70% of acute cerebrovascular events, or for strokes stratified by severity. Data for risk factors were conflicting because of small sample sizes and methodological heterogeneity.

**Added value of this study**

In a large 15-year prospective population-based longitudinal cohort of all acute cerebrovascular events, irrespective of age, with near-complete ascertainment and consistent diagnostic methods (in terms of procedures and personnel) for both cerebrovascular events and dementia, selection and attritional biases were substantially mitigated through the use of several methods of patient assessment. Rates of both pre-event and post-event dementia were highly dependent on clinical characteristics, with the incidence of new post-event dementia at 1 year varying substantially between patients with transient ischaemic attack and those with more severe stroke. Incidence was lowest after a first transient ischaemic attack among patients younger than 70 years (<1% 1 year after the event). Dementia was brought forward by 25 years for severe stroke, driven by an incidence during the first year after stroke that was 47 times higher than that in the general UK population; this effect was less pronounced after minor stroke (dementia brought forward by 4 years) and transient ischaemic attack (dementia brought forward by 2 years). Dementia risk was dependent on age, event severity, previous stroke, dysphasia, baseline cognition, low education, pre-morbid dependency, leucoaraiosis, and diabetes, but not on other vascular risk factors.

**Implications of all the available evidence**

Incidence of dementia in patients with transient ischaemic attack or stroke varies considerably depending on clinical characteristics. This information should inform counselling of patients and carers, targeted follow-up, and selection of patients for trials. Major stroke increases the incidence of dementia nearly 50 times in the short term, but the risk of dementia is substantially lower after transient ischaemic attack or minor stroke, particularly in the absence of other susceptibility factors. Our findings suggest a shared susceptibility to stroke and dementia, contributing to high rates of dementia before and after major stroke.

Dementia risk is probably related to individual clinical characteristics, and could depend on the acute lesion (eg, severity, location, sub-type), pre-existing lesion burden, vascular risk factors, and other determinants of susceptibility, such as education, white matter disease, and previous cognitive function.^1^, ^2^, ^8^, ^9^, ^10^ Data from epidemiological^1^, ^2^, ^11^ and genetic studies^12^ suggest a shared susceptibility to stroke and dementia, which might explain high rates of dementia before and after major stroke, but might also contribute to lower dementia risks after transient ischaemic attack or minor stroke despite a similar adverse vascular risk factor profile. We aimed to calculate the prevalence of pre-event dementia and the prevalence and incidence of post-event dementia in the longitudinal population-based cohort Oxford Vascular Study (OxVasc). We also sought to establish predictors of risk and to compare results from the OxVasc cohort with age-matched and sex-matched UK population-level dementia data.

## Methods

### Study background

OxVasc is a longitudinal population-based incidence cohort of all acute vascular events occurring within a defined population of 92 728, covered by around 100 primary care physicians in nine primary care practices in Oxfordshire, UK (appendix).^13^, ^14^, ^15^ An estimated 97% of the true study residential population is registered with a primary care practice; most unregistered people are young students. According to the index of multiple deprivation, the population is less deprived than the rest of England, but 22% of OxVasc districts rank in the lowest third nationally. The study area contains a mix of urban and rural populations. The OxVasc population is 94% Caucasian, 3% Asian, 2% Chinese, and 1% Afro-Caribbean.^16^ OxVasc was approved by the Oxfordshire clinical research ethics committee (CO.043). Written informed consent (or assent from relatives) was obtained for study interview and follow-up, including ongoing review of primary care and hospital records and death certificate data.

### Procedures

Consecutive patients with transient ischaemic attack or stroke were prospectively recruited from April 1, 2002, to March 31, 2012. Case ascertainment, including early deaths and transient ischaemic attack and stroke occurring in non-hospitalised, non-referred patients, was done with multiple methods, including daily searches of emergency departments, relevant wards, eye hospital attendances, bereavement office, and radiographic or vascular surgical investigations or procedures, and monthly searches of primary care practice diagnostic codes, referrals to acute and community hospitals, and death certificates and coroners' reports. Ascertainment of TIA and minor stroke, as well as major stroke, approached 100% of events reaching medical attention,^13^, ^14^, ^17^ thereby minimising selection biases in establishing dementia risk (appendix).^18^ Briefly, patients with transient ischaemic attack or minor stroke were referred directly by their primary care physician or the emergency department to dedicated daily OxVasc emergency clinics for acute management, whereas those with major stroke were admitted to an acute hospital (John Radcliffe Hospital, Oxford, UK) that covers the study population and were recruited as soon as possible after admission.

Patients were assessed by a study clinician as soon as possible after their transient ischaemic attack or stroke. Transient ischaemic attack was defined according to the National Institute of Neurological Disorders and Stroke criteria,^19^ and stroke according to the WHO criteria,^14^, ^15^ with review of all cases as soon as possible after presentation by the same senior neurologist (PMR) throughout the study.

For the present analyses, we included patients who had definite or probable transient ischaemic attack, as adjudicated by PMR, and excluded patients with possible transient ischaemic attacks. Patient data, including vascular risk factors, were collected by interview with a standardised form and supplemented by primary care records.^14^, ^15^ Premorbid functional status was assessed with modified Rankin and Barthel scores,^14^, ^15^ and stroke severity was assessed with the US National Institutes of Health Stroke Scale (NIHSS) score.^14^ Baseline brain and vascular imaging and other investigations were done as reported previously.^20^ Leucoaraiosis on brain imaging was defined as absent, mild, moderate, or severe according to standard scales.^21^

Multiple methods of follow-up were used to reduce attritional biases in identification of dementia, with follow-up to end study or death completed in more than 95% of patients (appendix).^22^ Follow-up interviews were done by trained nurses or study physicians at 1 month, 6 months, 1 year, and 5 years. Recurrent events were also identified between follow-up assessments by ongoing ascertainment of acute events. If follow-up at a clinic was not possible, patients were assessed at home or via telephone. Cognitive function was tested with the mini-mental-state-examination (MMSE)^23^ and Montreal Cognitive Assessment^24^ at face-to-face interviews and with the telephone version of the Montreal Cognitive Assessment and the Telephone Interview for Cognitive Status—modified during telephone follow-up.^25^, ^26^

Dementia was defined as pre-event or post-event according to whether the diagnosis was made before or after the index event, as described previously.^18^, ^22^ Briefly, pre-event dementia diagnoses were based on a baseline clinical assessment by a study physician and discussion with relatives or another informant, and the presence of any dementia diagnosis or related consultations and investigations in the primary care record. Diagnosis of pre-event dementia was done by a senior study physician with expertise in dementia (STP), who used the fourth edition of the Diagnostic and Statistical Manual of Mental Disorders (DSM-IV) criteria after review of the baseline clinical assessment and hand-searching of the entire primary care record, including individual consultations, clinic letters, and hospitalisation documentation.

Post-event dementia was diagnosed by STP according to the same methods (ie, with baseline and follow-up clinical and cognitive assessment data, supplemented by hand-searching of primary care records to death or 5-years' follow-up).^18^, ^22^ MMSE was done at each follow-up interview, and dementia was diagnosed if patients scored less than 24 on the MMSE and their score remained less than 24 for all subsequent follow-up visits.^18^, ^22^, ^27^, ^28^ In patients who had problems that interfered with testing (eg, dysphasia), could not complete testing (eg, because of blindness), were followed up by telephone, could not be tested at the study interview (eg, because of severe deafness),^29^ or who did not have a follow-up assessment, dementia was diagnosed on the basis of study records (when available) and hand-searching of primary care, hospital, and death records according to DSM-IV criteria, and the date of diagnosis was recorded. 18, 22, 29 For cases in which there was diagnostic uncertainty, all study and medical records information was reviewed, and uncertainty was resolved by discussion between STP and PMR.

### Statistical analysis

Baseline characteristics were near complete for demographic and clinical data, including event severity and vascular risk factors (appendix). Baseline characteristics were compared with ANOVA or χ^2^ test as appropriate. Analyses of dementia prevalence and incidence were stratified by severity of index event (transient ischaemic attack, stroke NIHSS <3, NIHSS 3–5, NIHSS 6–10, NIHSS >10), and by presence or absence of stroke before the index event. For age-stratified analyses (<65 years, 65–74 years, ≥75 years), only three event severity categories (TIA, NIHSS <3, and NIHSS ≥3) were used because of the small number of more severe events in each age category. In OxVasc, a minor stroke is defined as an NIHSS score of less than 3.^30^ Pre-event dementia prevalence estimates were calculated with the population proportion, and 95% CIs were derived with 1·96 times the SE of the estimate.

All analyses involving post-event dementia were done after exclusion of cases of pre-event dementia. Cumulative incidence and 95% CIs of post-event dementia were calculated by Kaplan-Meier methods, with censoring at death and loss to follow-up with and without further censoring for recurrent stroke on follow-up. If the exact dementia diagnosis date was unavailable, an estimated date was assigned after review of study and medical records, including information from informants. To account for the competing risk of death, we also calculated cumulative incidence with cumulative incidence competing-risk methods.^31^

We used the standardised prevalence ratio and standardised morbidity ratio to compare the prevalence and incidence, respectively, of dementia with published age-matched and sex-matched data for the UK population aged 65 years or older.^32^, ^33^ The standardised prevalence ratio is the ratio of observed prevalence to expected prevalence. The standardised morbidity ratio is the ratio of the observed incidence per 1000 person-years to the expected incidence per 1000 person-years, calculated by multiplying age-specific person-years of follow-up by the corresponding age-specific and sex-specific incidence of dementia in the UK. The expected prevalence and incidence of dementia were calculated for the sample by stratifying by sex and then according to the age bands used to report UK population data (ie, 65–69, 70–74, 75–79, 80–84, and ≥85 years).^32^, ^33^ The results obtained for each age-specific and sex-specific strata were then added to obtain the expected rates for the total sample.

Estimates for the number of years by which dementia was brought forward by cerebrovascular events of differing severity were obtained by visually comparing the plots for age-specific expected prevalence in the UK population with the observed age-specific prevalence in OxVasc survivors at 1 year, as described previously.^3^ Associations between baseline clinical characteristics (appendix) and pre-event dementia were established by binary logistic regression. For the association between baseline clinical characteristics and 5-year risk of new post-event dementia, we used Cox regression adjusted for age, sex, and education to generate hazard ratios (HRs). For post-event dementia, analyses were further adjusted for event severity (ie, according to NIHSS score). Because death is a competing risk for dementia (ie, it precludes the occurrence of dementia), we did exploratory analyses with competing risks regressions: we used cumulative incidence function covariate analysis, which were adjusted for age, sex, and education.^31^ From these regressions, we generated subdistribution HRs for comparison. Subdistribution HRs are thought to be more informative than standard HRs about risk or prognosis, whereas standard (ie, cause-specific) HRs are thought to be better indicators of cause.^34^

Risk factors were defined as markers of cerebral susceptibility or reserve if consistent with the concept of cerebral reserve (ie, resilience against age-related and disease-related change), or if they were known to be associated with dementia in non-stroke populations.^8^, ^9^, ^10^ All analyses were done in Stata (version 13).

### Role of the funding source

The study funders had no role in study design; data collection, analysis, or interpretation; or the writing of the report. The corresponding author had full access to all data in the study and had final responsibility for the decision to submit for publication.

## Results

Of 2305 patients recruited (mean age 74·4 years [SD 13·0]), 1133 (49%) were men and 1172 (51%) were women, 688 (30%) had transient ischaemic attacks, and 1617 (70%) had strokes (1482 ischaemic strokes and 135 primary intracerebral haemorrhages). Patients with major stroke (ie, NIHSS ≥3) were older and less educated and more likely to have premorbid dependency, previous strokes, atrial fibrillation, and baseline cognitive impairment than those with transient ischaemic attack or minor stroke (appendix).

**Figure 1:**
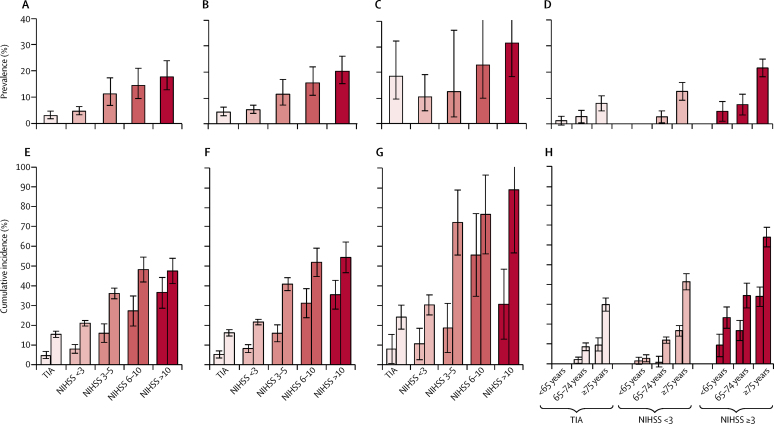
Prevalence of pre-event dementia in patients without previous stroke (A), all patients (B), and patients with previous stroke (C), by stroke severity, and in all patients stratified by age and stroke severity (D); and cumulative incidence of dementia, 1 year and 5 years post-event, in patients without previous stroke (E), all patients (F), and patients with previous stroke (G), by stroke severity, and in all patients stratified by age and stroke severity (H) The event is the index transient ischaemic attack or stroke. Patients with pre-event dementia were excluded from estimates of the cumulative incidence of post-event dementia. In (E)–(H), each pair of bars represents the 1-year (left) and 5-year (right) cumulative incidence of dementia. Error bars represent 95% CIs. Note that estimates differ slightly from those shown in figure 2 because the estimates shown here were derived from lifetable analyses. TIA=transient ischaemic attack. NIHSS=US National Institutes of Health Stroke Scale.

Pre-event dementia was diagnosed in 225 patients (mean age 83·3 years [SD 7·9]). Its prevalence was substantially lower in patients with transient ischaemic attack than in those with stroke (4·9% [95% CI 3·4–6·9] *vs* 11·8%; [10·2–13·6]; figure 1; appendix). Prevalence of pre-event dementia was substantially higher among people with a history of stroke before the index event than among those without such a history both for people with transient ischaemic attack (18·5% [95% CI 9·5–32·3] *vs* 3·5% [2·2–5·3]; p<0·0001) and those with stroke (23·4% [17·3–31·0] *vs* 10·1% [8·5–11·9]; p<0·0001; appendix). There was a clear stepwise association between increasing severity of the index stroke and the prevalence of pre-event dementia, ranging from a prevalence of 6·8% (95% CI 5·4–9·0) among patients with an NIHSS score less than 3 to 20·6% (15·8–26·5) among those with an NIHSS greater than 10 (p<0·0001 for the overall association; figure 1; appendix). The stepwise relationship was maintained within groups stratified by age, ranging from 0·6% (95% CI 0·1–3·5) in patients with transient ischaemic attack without previous stroke before age 65 years, to 27·0% (19·5–36·5) in patients with stroke (NIHSS score >3) who had previous strokes after age 75 years (figure 1).

Compared with age-matched and sex-matched UK population data, pre-event dementia prevalence was not significantly increased in patients with transient ischaemic attack (standardised prevalence ratio 0·7 [95% CI 0·5–1·0]) or minor stroke (1·1 [0·8–1·4]) but was higher in patients with more severe strokes; the relative prevalence reached 1·9 (1·5–2·5) in stroke (NIHSS>10; table 1; appendix).

**Table 1:** Pre-event dementia prevalence, post-event dementia incidence (after exclusion of pre-event dementia), and point prevalence of any dementia 1 year and 5 years after index event relative to age-matched and sex-specific UK population prevalence and incidence of dementia

		**SPR for pre-event dementia (95% CI)**	**SMR for incident dementia 1 year after index event (95% CI)**	**SMR for incident dementia 1–5 years after index event (95% CI)**	**SPR for any dementia 1 year after index event (95% CI)**	**SPR for any dementia 5 years after index event (95% CI)**
Transient ischaemic attack		0·7 (0·5–1·0)	3·5 (2·5–4·8)	1·5 (1·1–2·0)	1·2 (0·9–1·6)	1·4 (1·0–1·9)
All stroke		1·4 (1·2–1·6)	10·0 (8·7–11·5)	2·7 (2·2–3·2)	2·7 (2·3–3·1)	3·2 (2·7–3·7)
NIHSS score						
	<3	1·1 (0·8–1·4)	5·8 (4·4–7·5)	2·2 (1·7–2·8)	1·5 (1·1–2·0)	2·1 (1·6–2·7)
	3–5	1·4 (1·0–1·9)	9·1 (6·6–12·3)	2·7 (1·9–3·7)	2·4 (1·8–3·2)	4·0 (3·0–5·2)
	6–10	1·6 (1·1–2·2)	25·7 (18·6–34·4)	5·5 (3·1–8·9)	4·0 (2·8–5·6)	4·4 (2·9–6·4)
	>10	1·9 (1·5–2·5)	47·3 (35·9–61·2)	6·5 (2·6–13·4)	8·0 (6·0–10·4)	6·5 (4·1–9·8)

The event is the index transient ischaemic attack or stroke. We used SPRs and SMRs to compare prevalence and incidence of dementia in our cohort with published age-matched and sex-matched data available for the UK population aged ≥65 years (appendix).^32^, ^33^ SPRs were calculated by dividing the observed prevalence by expected prevalence. SMRs are the observed incidence per 1000 person-years divided by the expected incidence per 1000 person-years, and were calculated by multiplying age-specific and sex-specific person-years of follow-up by the corresponding UK age-matched and sex-specific dementia incidence. SPR=standardised prevalence ratio. SMR=standardised morbidity ratio. NIHSS=US National Institutes of Health Stroke Scale.

**Figure 2:**
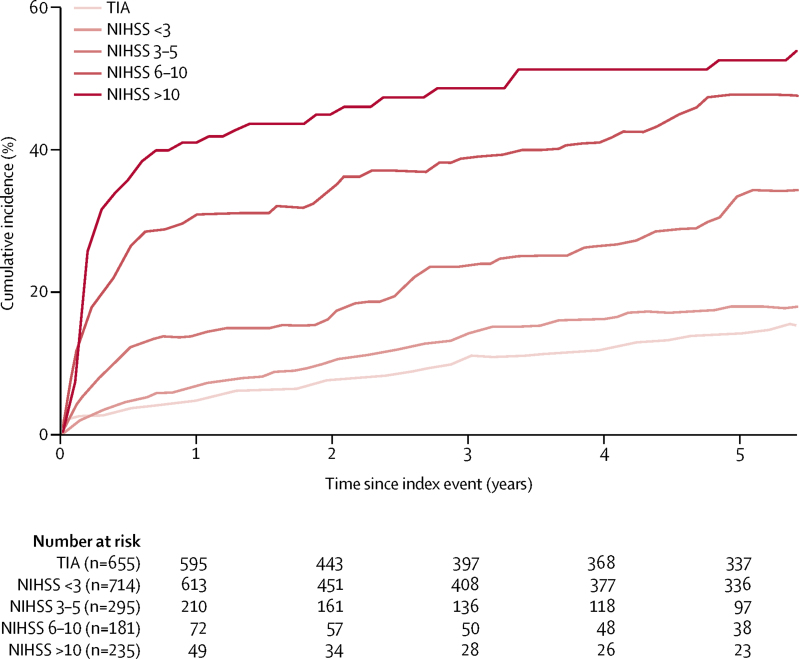
Kaplan-Meier curves of cumulative incidence of post-event dementia up to 5-years' follow-up, stratified by event severity The event is the index transient ischaemic attack or stroke. Patients with and without previous stroke were included, but those with pre-event dementia were excluded. TIA=transient ischaemic attack. NIHSS=US National Institutes of Health Stroke Scale.

Of the 2080 patients without pre-event dementia, 1982 (95%) were followed up to the end of study or death. Assent or consent to follow-up could not be obtained for 42 patients, and 56 patients moved out of the study area and could not be contacted (30 of these 56 patients were followed up to at least 1 year). 725 (35%) died overall: 141 (22%) of 655 patients with transient ischaemic attack, 186 (26%) of 714 patients with stroke with an NIHSS score of less than 3, 116 (39%) of 295 with stroke with an NIHSS score of 3–5, 106 (59%) of 181 with stroke with an NIHSS score of 6–10, and 176 (75%) of 235 with an NIHSS score of greater than 10 (appendix). During 7721 patient-years of follow-up (median 4·2 years [IQR 1·5–5·5]), 432 patients developed dementia (mean age at diagnosis 82·1 years [SD 8·7]). The 5-year cumulative incidence of new post-event dementia was 16·2% (95% CI 14·6–17·8) after transient ischaemic attack and 33·1% (31·7–34·5) after stroke (log rank p<0·0001; Figure 1, Figure 2; appendix). However, the incidence was higher (overall p for the association of previous stroke with dementia for the whole cohort=0·006) overall in patients with previous stroke than in those without (24·2% [95% CI 18·1–30·3] *vs* 15·4% [13·8–17·0] in patients with an index transient ischaemic attack and 47·6% [40·7–54·5] *vs* 30·4% [29·0–31·8] in patients with an index stroke; figure 1; appendix). 5-year cumulative incidence of new post-event dementia was also substantially higher in those with either previous or recurrent strokes than in those with neither (23·4% [18·5–28·9] *vs* 15·1% [13·5–16·7] among patients with an index transient ischaemic attack, and 49·8% [46·2–53·4] vs 26·6% [25·0–28·2] after index stroke).

As with pre-event dementia, we noted a stepwise association between severity of the index stroke and post-event dementia (p<0·0001), particularly at 1-year follow-up, with incidence of 5·2% (95% CI 3·4–7·0) after transient ischaemic attack, 8·2% (6·2–10·2) after minor stroke, and 34·4% (29·7–41·5) after severe stroke (Figure 1, Figure 2; appendix). Results were qualitatively similar for ischaemic stroke and primary intracerebral haemorrhage, although the number of patients with primary intracerebral haemorrhage was small (appendix). Even among patients with minor stroke, dementia incidence increased from NIHSS scores of 0 to scores of 2 (appendix). The relationship between event severity and post-event dementia was also maintained after stratification by age and previous stroke (figure 1; appendix), with a 5-year cumulative incidence ranging from 0% before age 65 years and 3·1% (95% CI 0·0–4·7) after transient ischaemic attack without previous stroke before age 70 years, to 81·5% (68·4–94·6) after major stroke (NIHSS≥3) with previous stroke after age 75 years.

**Figure 3:**
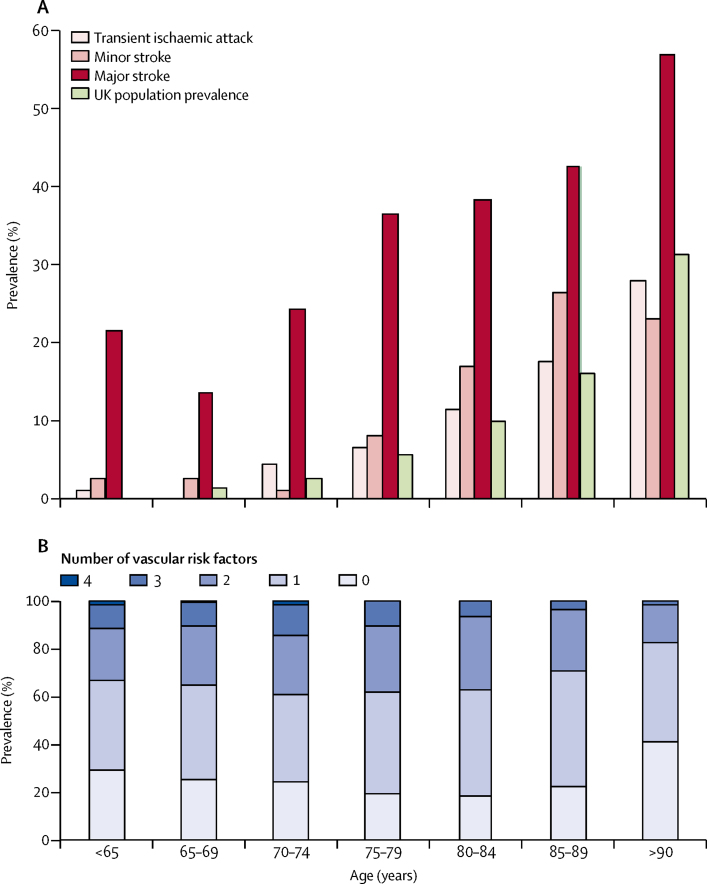
Prevalence of any dementia in patients 1 year after transient ischaemic attack or stroke and in the UK general population (A), and baseline prevalence of vascular risk factors (B), by age group The event is the index transient ischaemic attack or stroke. (A) Patients with pre-event or post-event dementia are included. Minor stroke was defined as a US National Institutes of Health Stroke Scale score of less than 3, whereas major stroke was defined as a score of 3 or higher. UK population prevalence data are age-matched and sex-matched data calculated from UK population estimates from the Medical Research Council Cognitive Function and Ageing Study.^12^, ^17^ (B) Vascular risk factors included were hypertension, diabetes, hypercholesterolaemia, and current smoking.

Of the 432 patients who developed post-event dementia before 5 years, 219 (51%) were diagnosed within the first year (Figure 1, Figure 2; appendix). The proportion diagnosed within the first year was higher among patients with severe stroke (60 [86%] of 70) than among those with minor stroke (48 [39%] of 124) or transient ischaemic attack (27 [32%] of 85). Compared with UK population data for those aged 65 years or older, the incidence of post-event dementia in the first year after the index event was higher for all categories of cerebrovascular event, with age-adjusted and sex-adjusted standardised morbidity ratios of 3·5 (95% CI 2·5–4·8) after transient ischaemic attack, 5·8 (4·4–7·5) after minor stroke, and 47·3 (35·9–61·2) after severe stroke. After the first year, the incidence in our cohort was still substantially higher than that in the UK general population, but not to the same extent (table 1; appendix). By contrast, owing to high rates of mortality associated with dementia, the standardised prevalence ratio for any dementia in survivors at 1-year follow-up (figure 3, table 1; appendix) was only 1·2 (0·9–1·6) after transient ischaemic attack and 1·5 (1·1–2·0) after minor stroke. However, it was substantially increased after severe stroke (8·0 [6·0–10·4]; table 1). Overall, for patients aged 70–75 years at the time of their event, the prevalence of dementia in 1-year survivors was brought forward by approximately 2 years in those who had transient ischaemic attacks (ie, the prevalence of dementia in 1-year survivors was roughly the same as that in patients 2 years older without transient ischaemic attack), 4 years in those who had minor strokes, and 25 years in those who had severe major stroke (ie, NIHSS score >10) compared with the age-matched and sex-matched general population (figure 3).

Baseline characteristics associated with pre-event and new post-event dementia were broadly similar (table 2; appendix). Significant predictors of post-event dementia after adjustment for age, sex, and education included age, event severity, previous stroke, dysphasia, baseline cognition, low education, pre-morbid dependency, leucoaraiosis, and diabetes (table 2). Other vascular risk factors (including hypertension, atrial fibrillation, hyperlipidaemia, and vascular disease) were not predictive of post-event dementia (table 2), as was also evident from their similar prevalence in transient ischaemic attack and minor stroke versus major stroke, despite the very different risk of dementia associated with these events (appendix). The same disconnection was also evident with regard to age, whereby young patients had high levels of vascular risk factors and yet much lower risks of dementia compared with older patients (figure 3). Subdistribution HRs were qualitatively similar to the (cause-specific) HRs for post-event dementia (appendix).

**Table 2:** Baseline characteristics associated with pre-event and post-event dementia at 5-year follow-up (after exclusion of pre-event dementia)

		**Pre-event dementia (adjusted for age, sex, and education)**		**Post-event dementia (adjusted for age, sex, and education)**		**Post-event dementia (adjusted for age, sex, education, and severity of event)**	
		Odds ratio (95% CI)	p value	Hazard ratio (95% CI)	p value	Hazard ratio (95% CI)	p value
Susceptibility factors							
	Age per year	1·09 (1·07–1·11)	<0·0001	1·09 (1·07–1·10)	<0·0001	1·08 (1·07–1·10)	<0·0001
	Age ≥75 years	6·13 (3·91–9·62)	<0·0001	4·69 (3·71–5·92)	<0·0001	4·58 (3·62–5·80)	<0·0001
	Male sex	0·97 (0·69–1·35)	0·85	0·85 (0·70–1·03)	0·10	0·91 (0·75–1·11)	0·37
	Low education*	1·25 (0·87–1·79)	0·23	1·69 (1·36–2·12)	<0·0001	1·63 (1·30–2·05)	<0·0001
	Pre-morbid modified Rankin Scale score ≥3	11·07 (7·56–16·21)	<0·0001	1·81 (1·43–2·28)	<0·0001	1·63 (1·29–2·05)	<0·0001
	Pre-morbid Barthel Index score <20	7·65 (5·03–11·64)	<0·0001	1·60 (1·28–2·00)	<0·0001	1·50 (1·20–1·87)	<0·0001
	Leucoaraiosis (moderate or severe)†	1·61 (1·14–2·29)	0·01	1·45 (1·19–1·77)	<0·0001	1·49 (1·22–1·82)	<0·0001
Current vascular risk profile							
	Treated hypertension	0·77 (0·56–1·08)	0·13	1·11 (0·91–1·37)	0·30	1·02 (0·83–1·25)	0·88
	Post-event systolic blood pressure	..	..	1·00 (0·93–1·07)	0·91	1·00 (0·99–1·00)	0·51
	Post-event diastolic blood pressure	..	..	0·96 (0·89–1·02)	0·20	1·00 (0·99–1·00)	0·26
	Diabetes	1·90 (1·25–2·88)	0·003	1·54 (1·19–2·00)	0·001	1·53 (1·18–1·98)	0·001
	Treated hyperlipidaemia	0·88 (0·61–1·27)	0·48	1·04 (0·84–1·27)	0·20	1·02 (0·83–1·25)	0·88
	Total cholesterol	0·92 (0·77–1·09)	0·31	0·95 (0·86–1·05)	0·29	0·95 (0·86–1·04)	0·29
	Atrial fibrillation	1·23 (0·86–1·75)	0·27	1·24 (0·99–1·55)	0·07	1·09 (0·87–1·37)	0·45
	Myocardial infarction	1·36 (0·88–2·11)	0·17	1·17 (0·88–1·55)	0·29	1·15 (0·87–1·53)	0·33
	Angina	1·41 (0·97–2·04)	0·08	0·91 (0·71–1·18)	0·49	0·98 (0·76–1·26)	0·85
	Peripheral arterial disease	1·28 (0·75–2·21)	0·37	1·26 (0·90–1·78)	0·19	1·27 (0·90–1·79)	0·18
	Current smoker	1·25 (0·70–2·23)	0·46	1·26 (0·90–1·74)	0·18	1·15 (0·83–1·60)	0·39
Low baseline cognitive score‡		19·90 (11·07–35·76)	<0·0001	4·34 (3·48–5·42)	<0·0001	3·22 (2·53–4·10)	<0·0001
Stroke lesion burden or location							
	Previous transient ischaemic attack (ie, before index event)	1·04 (0·65–1·64)	0·88	1·03 (0·79–1·36)	0·82	1·02 (0·78–1·34)	0·89
	Previous stroke (ie, before index event)	2·70 (1·84–3·94)	<0·0001	1·44 (1·11–1·86)	0·006	1·20 (0·93–1·56)	0·17
	Index transient ischaemic attack	1	<0·0001	1	<0·0001		<0·0001
	Index ischaemic stroke	2·38 (1·56–3·65)	..	2·53 (1·99–3·22)	..	1·71 (1·32–2·21)	..
	Index primary intracerebral haemorrhage	1·46 (0·58–3·69)	..	4·51 (2·84–7·17)	..	2·59 (1·60–4·19)	..
NIHSS							
	<3	1·45 (0·92–2·29)	..	1·57 (1·19–2·07)	<0·0001	..	..
	3–5	2·20 (1·34–3·61)	..	2·72 (2·01–3·67)	<0·0001	..	..
	6–10	3·15 (1·90–5·20)	..	4·88 (3·53–6·75)	<0·0001	..	..
	>10	3·54 (2·23–5·61)	..	7·67 (5·55–10·59)	<0·0001	..	..
	Per point	1·05 (1·03–1·08)	<0·0001	1·12 (1·10–1·13)	<0·0001	..	..
Dysphasia		1·69 (1·16–2·47)	0·002	3·26 (2·63–4·04)	<0·0001	1·84 (1·42–2·38)	<0·0001
Brain imaging (CT/MRI)§							
	Acute lesion	1·04 (0·71–1·51)	0·83	1·84 (1·50–2·26)	<0·0001	1·33 (1·07–1·64)	0·01
	Old lesion	1·83 (1·21–2·76)	0·001	1·09 (0·85–1·41)	0·49	0·89 (0·69–1·15)	0·39
	Multiple lesions	1·33 (0·59–3·01)	0·83	1·91 (1·11–3·28)	0·020	1·78 (1·16–2·71)	0·008

The event is the index transient ischaemic attack or stroke. NIHSS=US National Institutes of Health Stroke Scale.

* 12 years or less of education.

† Defined according to Simoni et al.^21^

‡ Score of less than 24 on the mini-mental state examination (or acute confusion if patient is untestable).

§ Data were available for ischaemic lesions only, which were classified as acute or old after review of the baseline brain CT or MRI in conjunction with the radiology report by the OxVASC study team; patients with multiple lesions could have a mixture of acute and old lesions.

Although primary intracerebral haemorrhage seemed to be more strongly associated than ischaemic stroke with new post-event dementia (HR 4·51 [95% CI 2·84–7·17] *vs* 2·53 [1·99–3·22]), this difference was less pronounced after adjustment for stroke severity (2·59 [1·60–4·19] *vs* 1·71 [1·32–2·21]; table 2). Examination of the association between stroke subtypes and risk of post-event dementia after exclusion of cases with transient ischaemic attack showed no association with primary intracranial haemorrhage (HR 1·49 [95% CI 0·97–2·28]; p=0·07 for primary intracranial haemorrhage vs ischaemic stroke) after adjustment for event severity and demographic factors (data not shown). In general, factors associated with dementia in ischaemic stroke and in primary intracerebral haemorrhage seemed to be broadly similar, but the number of patients with primary intracerebral haemorrhage was small (appendix).

238 patients who had remained dementia free after the initial index event had at least one stroke during follow-up. Recurrent stroke during follow-up increased the risk of new dementia (appendix), particularly during the first year after the recurrent event, and also in relation to increased stroke burden, both in terms of the severity of the recurrent event (HR 1·42 [95% CI 1·12–1·82]; p=0·005) and the severity of the previous index event (1·99 [1·55–2·54; p<0·0001), although the association with the severity of the recurrent event attenuated when both the severity of both the recurrent event and the index event were entered into the model (1·24 [0·95–1·61]; p=0·12 for the recurrent event and 1·99 [1·55–2·54]; p<0·0001 for the index event).

## Discussion

We found stepwise associations between the severity of the index cerebrovascular event and both pre-event and post-event dementia, irrespective of age, which were modified by previous and recurrent stroke and by markers of cerebral susceptibility or reserve. Variation in dementia risk was therefore substantial—with 5-year cumulative incidence ranging from 0% after transient ischaemic attack in patients aged younger than 65 years to more than 80% in those aged 75 years or older with major recurrent stroke—highlighting the importance of individualised prognostication. The particularly high risk in patients with the greatest stroke burden is consistent with direct effects of brain lesions on cognition, although shared susceptibility to stroke and dementia might also have a role in light of the association between event severity and pre-event dementia.

We (appendix) and others^6^ have previously reported that 25–50% of patients with transient ischaemic attack or minor stroke have some cognitive impairment during early follow-up, but few studies of risk of progression to dementia have been done. De Ronchi and colleagues^3^ reported that, in a volunteer population of older adults (mean age 73 years), the occurrence of stroke brought forward the onset of dementia by about 10 years. However, they did not stratify their data by age or by severity of event, and did not study the effect of transient ischaemic attack. We found that 5-year incidence of dementia is very strongly related to both age and severity of event, with very low incidence in younger patients who had transient ischaemic attacks or minor stroke, despite a high burden of vascular risk factors, although we were not yet able to examine longer-term risks (figure 3).

The effect of stroke severity and burden on risk of dementia was evident in all age groups, with severe stroke (ie, NIHSS score >10) bringing forward dementia by about 25 years, transient ischaemic attack bringing dementia forward by 2 years, and minor stroke by 4 years. Although the absolute risk of dementia was lower at younger ages than at older ages, the relative effect of major stroke compared with transient ischaemic attack or minor stroke was greatest in patients aged 64 years or younger in keeping with previous studies.^1^, ^35^ As expected, the prevalence of pre-event dementia was low in patients younger than 65 years, but approximately 20% of patients younger than 65 years who had a major stroke developed dementia during 5 years' follow-up.

Most dementia occurred in the first year after major stroke, whereas onset was more gradual after minor stroke. The effect of recurrent events was consistent with the concept of stepwise decline or multi-infarct dementia first proposed by Hachinski.^36^ Dementia risk in the first year after a stroke was higher in our study than in previous studies in which routine health service coding data were used, or in which analyses were restricted to those surviving at 3–6 months or to those with their first ever stroke.^1^, ^35^ A higher early versus later risk of dementia was also noted after transient ischaemic attack, although to a lesser extent than after stroke, which might be expected because 20–30% of patients with a transient ischaemic attack meeting the WHO criteria have some infarction on brain imaging.^37^ This apparent temporal effect of the stroke lesion is consistent with previous studies,^1^, ^30^, ^38^ and could be mediated by circuit disruption and diaschisis,^39^, ^40^, ^41^ or by inflammatory mediators from the acute vascular lesion or systemic complications.^42^

Post-event dementia risk was also related to previous susceptibility or reserve,^43^, ^44^ particularly at older ages, when even minor events can trigger dementia.^45^ As expected, a short baseline cognitive screen was also predictive of dementia risk,^5^, ^45^, ^46^ probably reflecting both previous cognition and lesion effects, although testing can be difficult early after major stroke.^29^ In combination with age, lesion burden and location, and other markers of susceptibility, clinical assessments should allow for reliable individual risk prediction to counsel patients, target follow-up, and recruit to trials.

Vascular risk profile, other than diabetes, was not associated with pre-event or post-event dementia, findings that were again consistent both with Hachinski's view that vascular factors contribute to dementia through cerebral infarcts and white matter changes more often than they cause dementia directly,^36^ and with the findings of negative trials 47, 48, 49 of the effect of intensive management of vascular risk factors on dementia risk after transient ischaemic attack and stroke. We did, however, note a fairly strong association between diabetes and dementia, consistent with the results of previous studies.^1^, ^44^

Strengths of our study include the population-based longitudinal design, high rates of ascertainment of transient ischaemic attack and minor stroke,^13^, ^14^ consistent diagnostic methods (in terms of both procedures and personnel) throughout the study period, and low loss to follow-up.^22^ However, our study also had some limitations. First, we did not formally test for pre-morbid mild cognitive impairment throughout the study. Second, pre-event dementia diagnosis was necessarily retrospective, but the use of multiple sources should have minimised under-ascertainment. Third, low prevalence of pre-event dementia in patients with transient ischaemic attack could have been caused by patients or carers not recognising or reporting transient symptoms in patients with dementia. Fourth, use of the MMSE could have resulted in underdetection of vascular dementia or overdiagnosis in less educated people. However, the MMSE is reliable for diagnosis of dementia in stroke and non-stroke populations,^27^, ^28^ and we were careful to avoid spurious diagnosis based on low scores caused by testing difficulties, including dysphasia.^29^ Fifth, one clinician rather than a consensus panel diagnosed dementia. However, several sources were used to ascertain dementia, which substantially mitigated selection and attritional biases that otherwise could have had a major effect on the measured dementia incidence and prevalence.^18^, ^22^ Furthermore, we excluded cases of transient cognitive impairment or so-called pseudo-dementia associated with depression. Sixth, because we used data from the population-based Medical Research Council Cognitive Function and Ageing Study^32^, ^33^ (MRCCFAS) to calculate relative dementia incidence and prevalence, differences in study methods could have introduced bias, and we could not adjust for possible differences in covariates other than age and sex. However, cognitive screening was similar in the two studies and selective attrition during follow-up was taken into account in both. Furthermore, although we used national MRCCFAS rates for comparison with our Oxfordshire data, there was little variation in age-specific or sex-specific rates by region in MRCCFAS.^32^, ^33^ Seventh, we did not examine associations of thrombolysis or thrombectomy with outcomes because these interventions were not established in the early period of recruitment. Finally, we did not report dementia subtype because clinical classification is challenging in older patients, and mixed pathology is the most common finding in neuropathological studies. Furthermore, most dementia occurring after stroke is coded as being of unspecified cause in routine practice.^35^

Our findings suggest that improvements in acute stroke care to reduce lesion severity might not only lessen immediate disability but also reduce the long-term risk of dementia. Further studies are therefore required to establish whether specific treatments, such as thrombectomy, reduce dementia risk.^50^, ^51^ By contrast, interventions aimed at reducing risk factors for dementia (other than those aimed at diabetes) beyond robust standard secondary prevention might have little short-term effect on dementia risk, particularly in older patients in whom the risk of adverse effects might be increased.
