# Supplementary material for: Incidence and prevalence of dementia associated with transient ischaemic attack and stroke: analysis of the population-based Oxford Vascular Study
Source: Lancet Neurol. 2019 Mar;18(3):248–58. doi: 10.1016/S1474-4422(18)30442-3 (PMC6390174; doi:10.1016/S1474-4422(18)30442-3)
Supplement: Supplementary appendix [file mmc1.pdf]

THE LANCET
Neurology

Supplementary appendix

This appendix formed part of the original submission and has been peer reviewed. We post it as supplied by the authors.

Supplement to: Pendlebury ST, Rothwell PM, for the Oxford Vascular Study. Incidence and prevalence of dementia associated with transient ischaemic attack and stroke: analysis of the population-based Oxford Vascular Study. *Lancet Neurol* 2019; **18:** 248–58.

Supplementary Appendix

Incidence and prevalence of dementia associated with TIA and stroke: rates and risk factors in a population-based cohort

Sarah T Pendlebury, Peter M Rothwell for the Oxford Vascular Study

Centre for Prevention of Stroke and Dementia, Nuffield Department of Clinical Neurosciences, John Radcliffe Hospital, and the University of Oxford

Correspondence to:

Sarah Pendlebury
Centre for Prevention of Stroke and Dementia
Level 6 West Wing
John Radcliffe Hospital
Oxford
OX3 9DU
Tel: +44 1865 231603
Fax: +44 1865 234639

Email: sarah.pendlebury@ndcn.ox.ac.uk

Supplementary Methods

OXVASC methodology

Study population

The Oxford Vascular Study (OXVASC) is a prospective, population-based cohort study of all incident acute vascular events in all territories (transient ischaemic attack, stroke, acute coronary and peripheral vascular events).[1-3]

During the period of the current substudy, the OXVASC study population consisted of all 92,728 individuals, irrespective of age, registered with 100 general practitioners (GPs) in nine general practices in Oxfordshire, UK. In the UK, general practices provide primary health care for registered individuals and hold a lifelong record of all medical consultations (from the National Health Service [NHS] and private health care), and details of treatments, blood pressure, and investigations. In Oxfordshire, an estimated 97% of the true residential population is registered with a general practice, with most non-registered individuals being young students. All participating practices held accurate age-sex patient registers, and allowed regular searches of their computerised diagnostic coding systems. The practices had all collaborated on a previous population-based study, for which they were originally selected to be representative of the urban and rural mix and the deprivation range of Oxfordshire as a whole.[4] Based on the index of multiple deprivation (IMD), the population was less deprived than the rest of England, but had a broad range of deprivation.
The OXVASC population is 94% white people, 3% Asian, 2% Chinese, and 1% Afro-Caribbean.[4] The proportion of whites is similar to that of the UK as a whole (88% white) and to many other western countries (Australia - 90%; France - 91%; Germany - 93.9%).

Case ascertainment

After a 3-month pilot study, the study started on April 1, 2002, and is ongoing. Ascertainment combined prospective daily searches for acute events (hot pursuit) and retrospective searches of hospital-care and primary-care administrative and diagnostic coding data (cold pursuit).

Hot pursuit was based on:

1. A daily (weekdays only), urgent open-access “TIA clinic” to which participating general practitioners (GPs) and the local accident and emergency department (A&E) send all individuals with suspected TIA or stroke whom they would not normally admit to hospital, with alternative on-call review provision at weekends. Patients too frail to attend are assessed at their residence by a study nurse or doctor.

2. Daily searches and case note review of admissions to the Emergency Assessment Unit, Medical Short Stay Unit, Coronary Care Unit and Cardiothoracic Critical Care Unit, Cardiology, Cardiothoracic, and Vascular Surgery wards, Acute Stroke Unit, Neurology ward and all other general wards when indicated.
3. Daily searches of the local A&E and eye hospital attendance registers.
4. Daily identification via the Bereavement Office of patients dead on arrival at hospital or who died soon after.
5. Daily searches of lists of all patients from the study population in whom a troponin-I level had been requested.
6. Daily assessment of all patients undergoing diagnostic coronary, carotid and peripheral angiography, angioplasty, stenting or vascular surgical procedures in any territory to identify both total burden of vascular invention and any potential missed prior acute events.

Cold pursuit procedures were:

1. Frequent visits to the study practices and monthly searches of practice diagnostic codes.
2. Monthly practice-specific list of all patients admitted to all acute and community NHS hospitals.
3. Monthly listings of all referrals for brain or carotid imaging studies performed in local hospitals.
4. Monthly reviews of all death certificates and coroners reports to review out-of-hospital deaths.
5. Practice-specific listings of all ICD-10 death codes from the local Department of Public Health.

Patients found on GP practice searches who have an event whilst temporarily out of Oxfordshire are included, but visitors who were not registered with one of the study practices are excluded. A study clinician assessed patients as soon as possible after the event in the hospital or at home. Informed consent was sought, if possible, or assent was obtained from a relative.

Data are collected using event-specific forms, for TIA and stroke, acute coronary syndrome or acute peripheral vascular events. Standardised clinical history and cardiovascular examination are recorded. Information recorded from the patient, their hospital records and their general practice records includes details of the clinical event, medication, past medical history, all investigations relevant to their admission (including blood results, electrocardiography, brain imaging and vascular imaging-duplex ultrasonography, CT-

angiography, MR-angiography or DSA) and all interventions occurring subsequent to the event.

If a patient died before assessment, we obtained an eyewitness account of the clinical event and reviewed any relevant records. If death occurred outside the hospital or before investigation, the autopsy result was reviewed. Clinical details are sought from primary care physicians or other clinicians on all deaths of possible vascular aetiology. In a previous study, only 3/823 interviewed patients reported previous vascular events that had not been ascertained using these multiple methods, thus the ascertainment rate is >99% of events presenting to medical attention.[3]

All surviving TIA and stroke patients are followed-up face-to-face at 1, 6, 12, 60 and 120 months after the initial event by a research nurse or physician and all recurrent vascular events were recorded together with the relevant clinical details and investigations. If face-to-face follow up is not possible, telephone follow-up is performed or enabled via the general practitioner. Cognitive function is tested using MMSE and MoCA at face-to-face interview and T-MoCA and TICSm on telephone follow-up.[5,6] All recurrent vascular events that presented to medical attention would also be identified acutely by ongoing daily case ascertainment within OXVASC. If a recurrent vascular event was suspected at a follow-up visit or referred by the GPs to clinic or admitted, the patient was re-assessed and investigated by a study physician.

Definitions of TIA/stroke events

Although new definitions for stroke and TIA have been suggested recently,[7,8] in order to enable comparison with previous studies, the classic definitions of TIA and stroke are used throughout.[9] A stroke is defined as rapidly developing clinical symptoms and/or signs of focal, and at time global (applied to patients in deep coma and to those with subarachnoid haemorrhage), loss of brain function, with symptoms lasting more than 24 hours or leading to death, with no apparent cause other than that of vascular origin.[9] A TIA is an acute loss of focal brain or monocular function with symptoms lasting less than 24 hours and which is thought to be caused by inadequate cerebral or ocular blood supply as a result of arterial thrombosis, low flow or embolism associated with arterial, cardiac or haematological disease.[4] All cases were reviewed as soon as possible after presentation by the same senior neurologist (PMR) throughout the study. For the current analyses, we included patients who had definite or probable TIA as adjudicated by PMR and excluded patients with possible TIA. Brain imaging was not used to define TIA. With the high rate (97%) of imaging or autopsy in OXVASC, strokes of unknown type were coded as ischaemic.

Dementia diagnosis

We examined issues around measured dementia diagnosis with reference to the OxVASC methodology in three previous publications in Stroke, specifically the impact of selection[10] and attrition[11] biases and problems interfering with cognitive testing.[12].

In OxVASC, we used multiple methods of follow-up which have been shown to substantially reduce attritional biases in identification of dementia in OxVASC.[11] Dementia was defined as pre- or post-event according to whether the diagnosis was made before or after the index event.[10] Pre-event dementia diagnosis was made using the following information: i) baseline clinical assessment by study physician and discussion with relatives or other informant; ii) any dementia diagnosis, and related consultations and investigations, where available, in the primary care record, with hand-searching of the entire record including individual consultations, clinic letters, and hospitalisation documentation. In many cases, diagnosis was recorded in hospital notes or clinic letters but not in the primary care diagnosis list. In other cases, the diagnosis was made by STP on the basis of cognitive and functional impairment apparent from hand-searching of the medical record including individual primary care consultations or clinical hospital physician, nursing and allied health care professional records.

In patients without pre-event dementia, post-event dementia was diagnosed by STP using the same methodology (i.e. using the baseline and follow-up clinical and cognitive assessment data, supplemented by hand-searching of primary care records to death or 5-year follow-up). MMSE and MoCA were done at each follow-up interview, and dementia was diagnosed if MMSE was <24 and remained <24 for all subsequent follow-ups in patients in whom cognitive testing was not affected by problems such as poor vision, hemiparesis or depression. A small number of subjects had a MoCA but no MMSE in whom none had dementia. In patients with incomplete testing or inability to perform a cognitive test at study interview (e.g. severe deafness) or with missing study follow-up assessment, dementia was diagnosed by STP on the basis of hand-searching of primary care, hospital and death records, based on DSM-IV criteria as described for pre-event dementia.[12]

Regarding date of dementia diagnosis, although study interview did not routinely occur between 1 and 5 years after the index event, some patients had data from a study interview during this period because of a recurrent event. In other patients, details of a dementia diagnosis made between 1 and 5-year follow-up were obtained at the 5-year study follow-up

and the date of diagnosis was obtained from medical records. For patients who did not have 5-year follow-up (eg because of death or drop-out between 1 and 5 years, untestability, telephone/email follow-up without a cognitive test), all available medical records were reviewed by STP. Where available, the exact date of diagnosis was recorded. If there was no clear date given in records, an approximate date of diagnosis was assigned based on review of study and medical records and information from informants where available.

We did not assess for functional impairment in patients diagnosed with dementia using the MMSE scores partly because it can be difficult attributing functional impairment to cognition versus physical disability in patients with cerebrovascular events. However, we performed sensitivity analyses to check whether this may have affected our results. Thirty-seven patients had low MMSE with a modified Rankin score of <2. In sensitivity analyses, removal of these patients had no significant effect on our findings and specifically, no impact on the relationship between event severity and dementia (HR=1.12 (1.10-1.13) per point increase in NIHSS vs 1.12 (1.10-1.13) adjusted for age, sex and education). Similarly, use of a lower cut-point (MMSE<20) in tested patients, did not change the relationship between event severity and post-event dementia (HR= 1.12 per point increase in NIHSS, p<0.0001).

For cases in which there was uncertainty (mainly in deciding whether cognitive impairment was sufficiently severe pre-event to be classed as pre-event dementia rather than progressing post-event to dementia), all study and medical records information was reviewed and resolved by discussion between STP and PMR.

Missing data

Data were complete for age and sex and clinical characteristics including event severity, vascular disease markers and vascular risk factors (Table S1). Missing data for education, premorbid dependency and disability, and baseline cognitive score are shown in Table S1. The majority of these missing data occurred in patients with early death including deaths in the community prior to any medical assessment or shortly after hospital admission and those who declined study interview. Missing cognitive data were also caused by untestability owing to patient factors (eg aphasia, reduced conscious level) largely occurring in major stroke as described previously.[12] Loss to follow-up before dementia diagnosis, death or end study was relatively low at 98/2305 (4%) and most commonly occurred when the patient moved out of area and could not be contacted (n=56) although 30/56 had follow-up to at least 1-year. Most other patients who moved away remained available to telephone follow-up.

Supplementary Figure S1

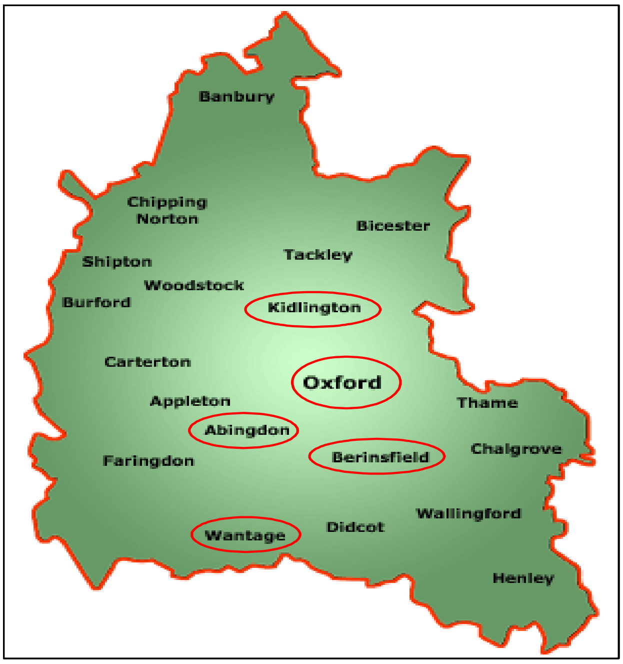

Figure S1. Schematic diagram of the county of Oxfordshire, UK and the towns in which the nine participating GP practices (circled) are located within the Oxford Vascular Study (OxVASC).

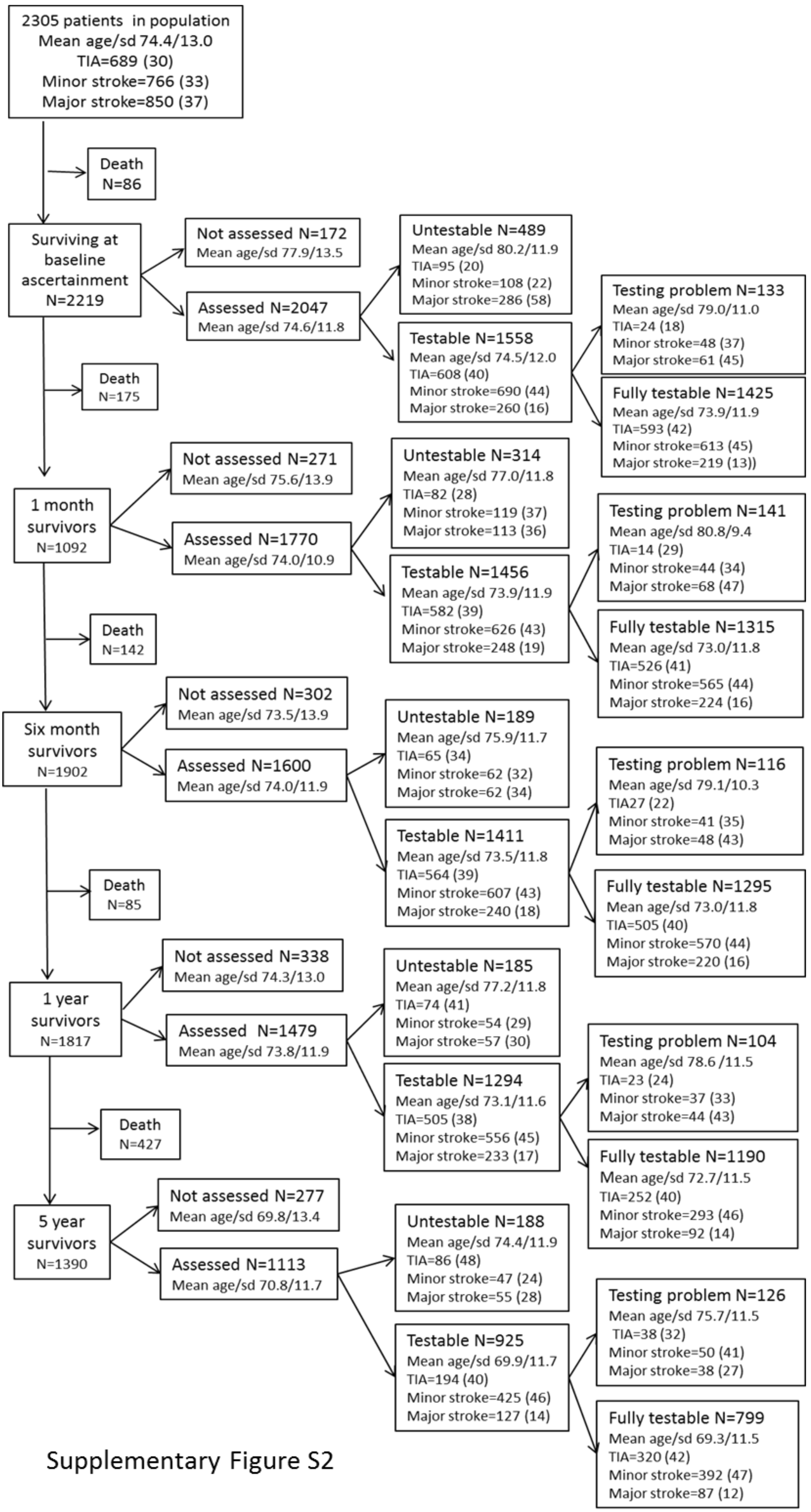

Supplementary Figure S2

Supplementary Figure S3.

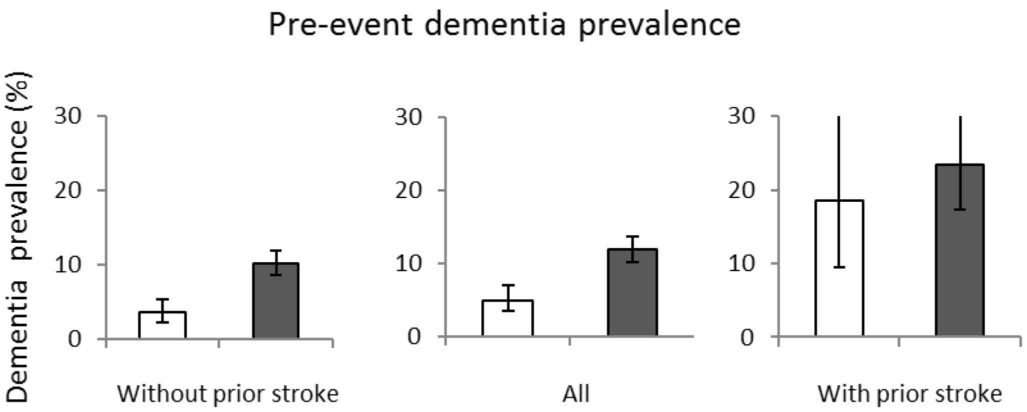

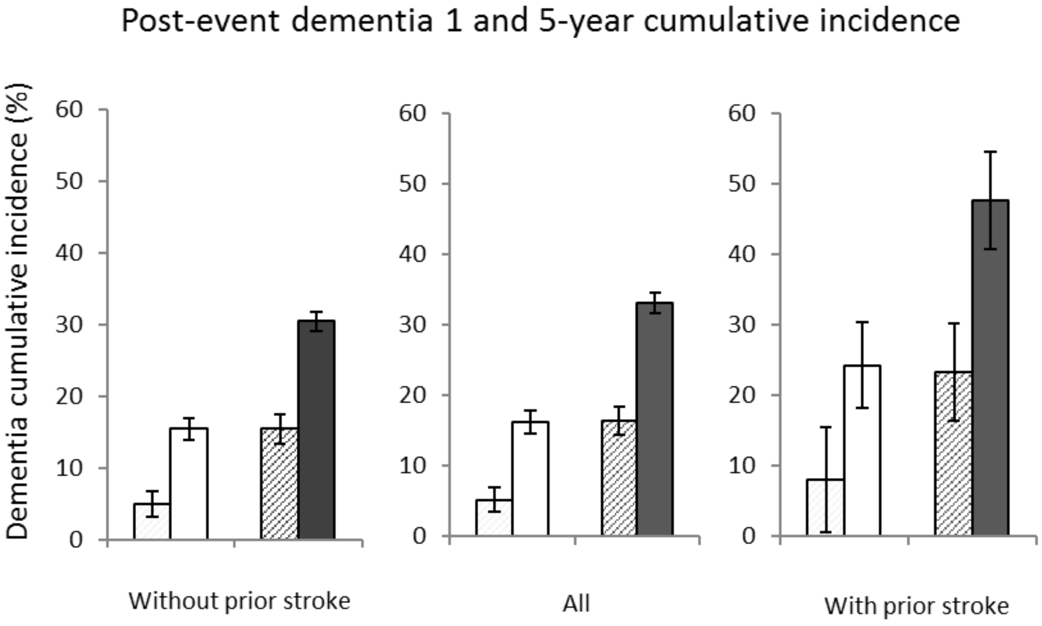

Figure S3. Pre-event dementia prevalence and post-event 1- (stippled bars) and 5-year (solid bars) cumulative incidence of dementia stratified by TIA (white bars) vs stroke (grey bars). Data are shown for patients without prior stroke, for all patients and for patients with prior stroke.

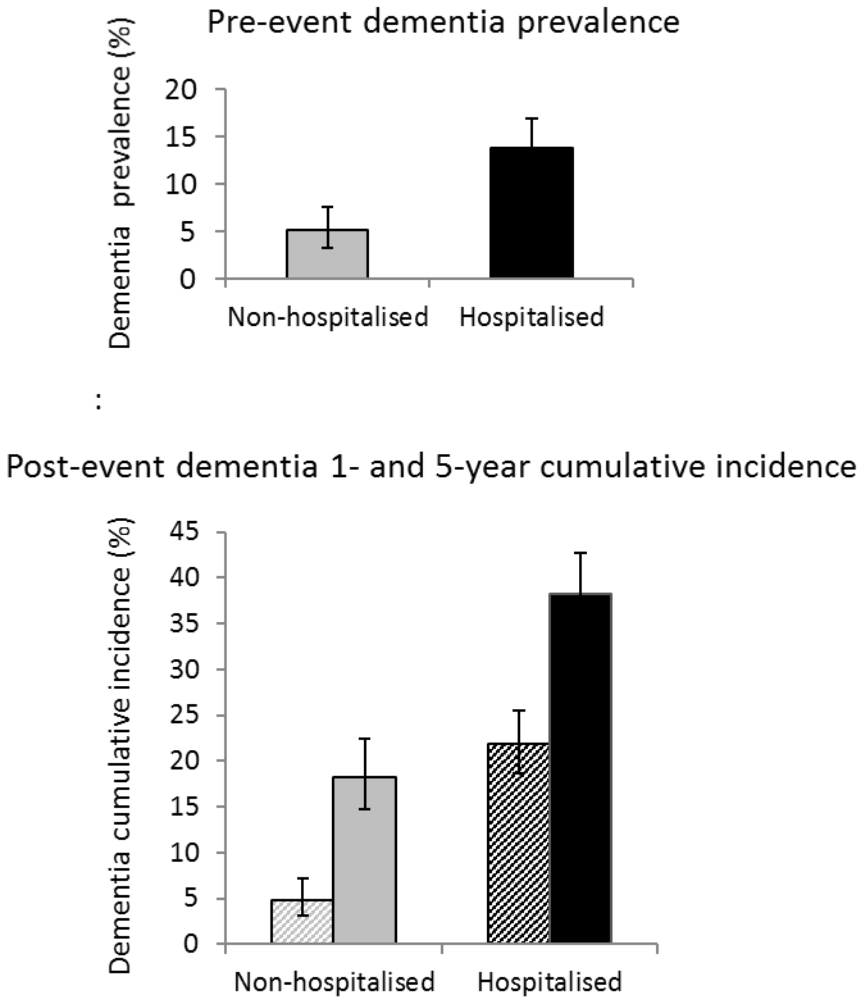

Figure S4.

Top: pre-event dementia prevalence in non-hospitalised stroke (NIHSS mean/sd=1.5/2.2, median (IQR) 1 (0,2)) vs hospitalised stroke patients (NIHSS mean/sd 7.4/7.2, median (IQR) 5 (2,11))

Bottom: Cumulative incidence of post-event dementia at 1- (stippled bars) and 5-years (solid bars) in non-hospitalised stroke (mean/sd NIHSS 1.5/2.1, median (IQR) 1 (0,2) vs hospitalised stroke patients (NIHSS mean/sd 7.1/7.0, median (IQR) 5 (2,10)).

Supplementary Figure S5

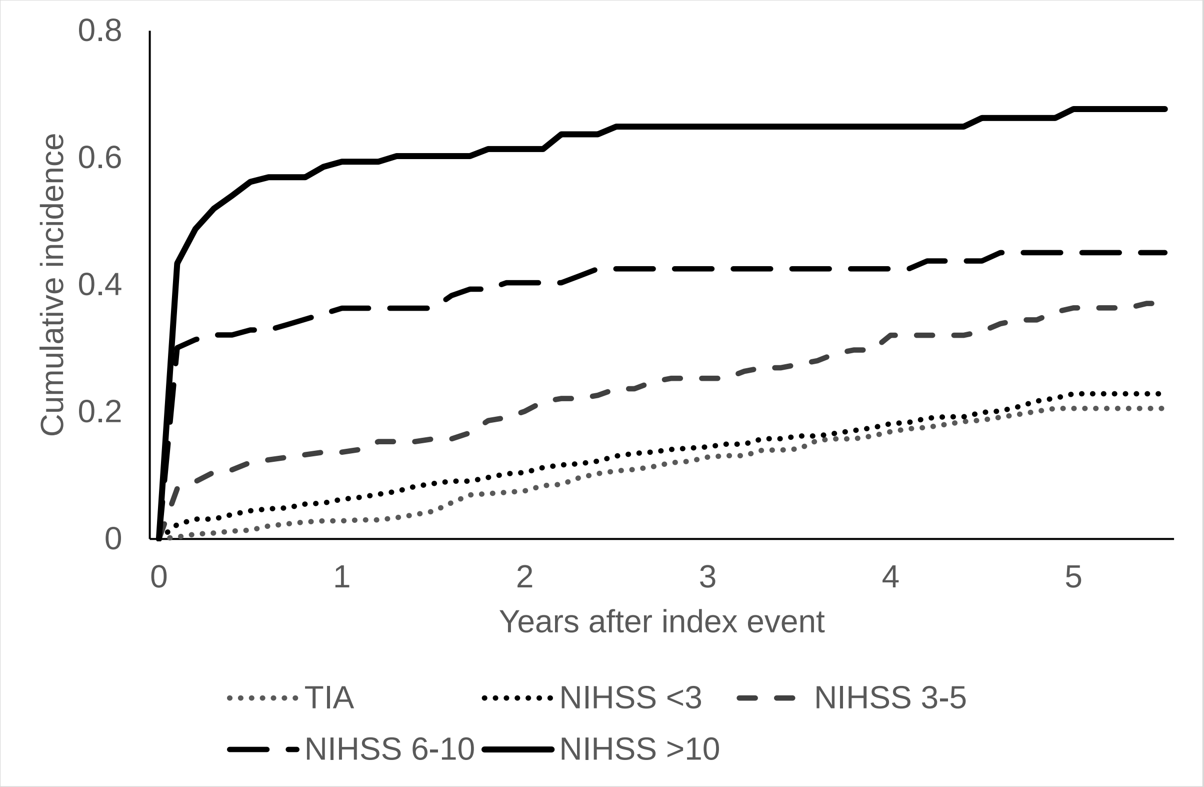

Figure S5

Kaplan-Meier curves showing time to death by severity of index event for the whole cohort of 2305 patients.

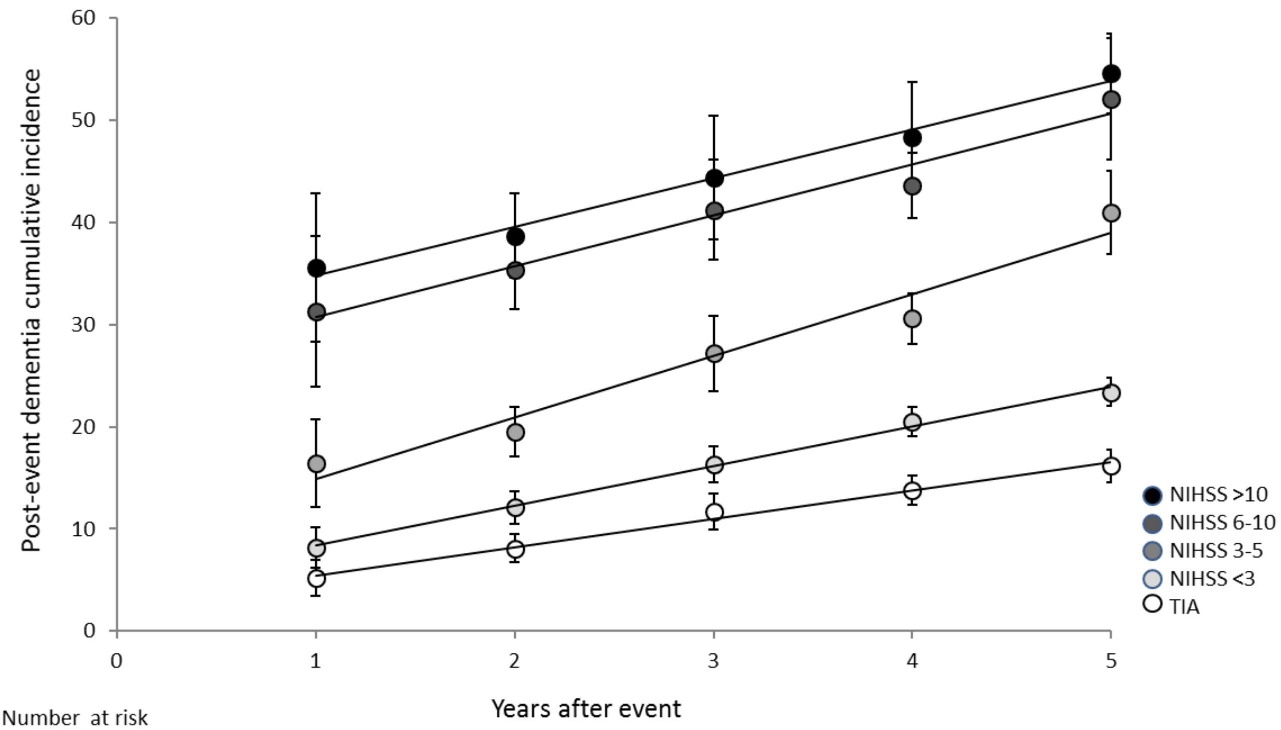

Number at risk					
TIA: 655	595	443	397	368	337
NIHSS<3: 714	613	451	408	377	336
NIHSS=3-5: 295	210	161	136	118	97
NIHSS=6-10: 181	72	57	50	48	38
NIHSS>10: 235	49	34	28	26	23

Figure S6.

Cumulative incidence of new post-event dementia (excluding pre-event dementia) for all patients (with and without prior stroke) to 5-years follow-up with 95% confidence limits, stratified by severity of event. Numbers are derived from the Kaplan-Meier curves shown in Figure 3, (main manuscript).

Supplementary Figure S7

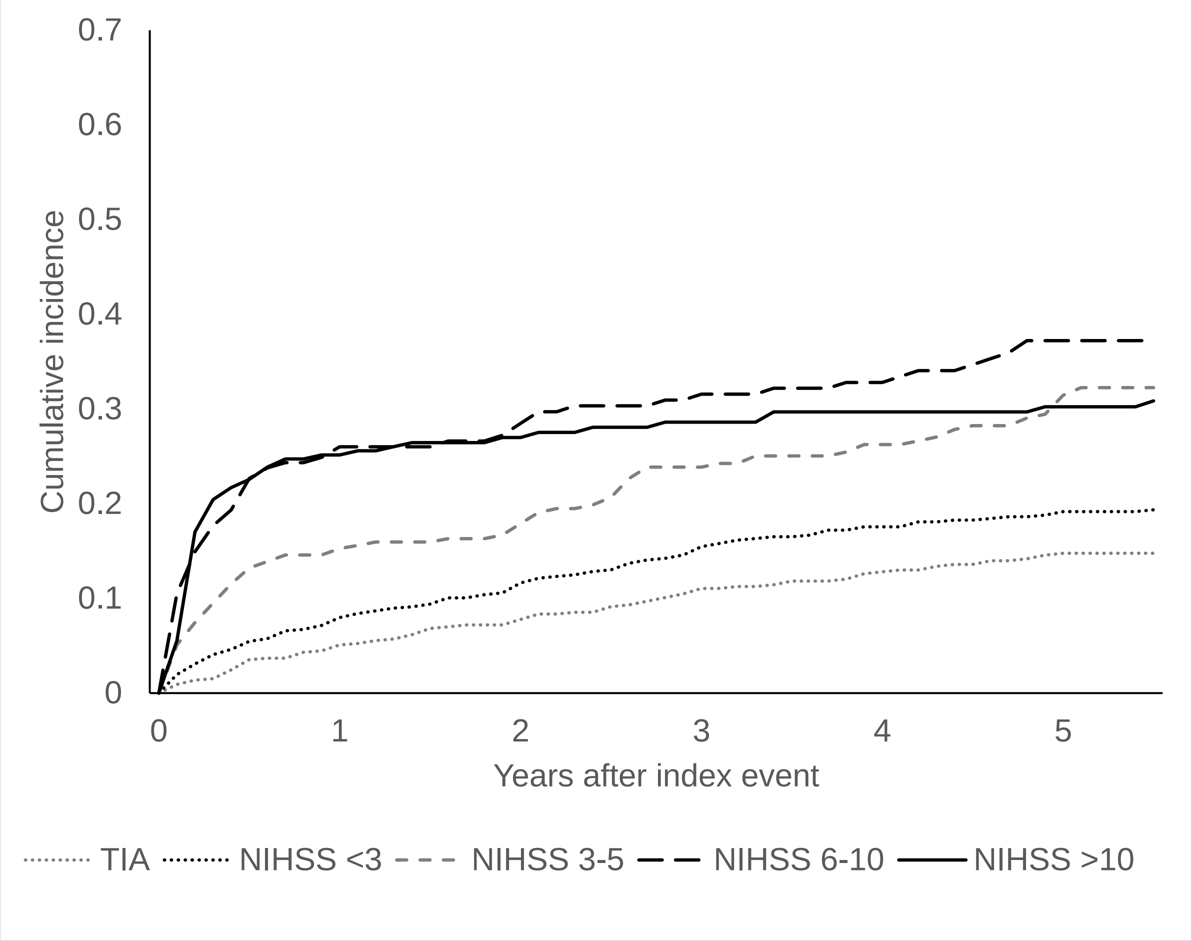

Figure S7. Given that death is a competing risk for dementia (i.e. it precludes its occurrence), we performed exploratory analyses showing the calculated cumulative incidence of post-event dementia using Cumulative Incidence Competing Risk methods[14,15] The graphs show that using Competing Risk methods, the cumulative incidence of dementia associated with severe stroke (NIHSS>10) is reduced compared to that obtained using Kaplan-Meier methods (Figure 2, main manuscript and Supplementary Appendix Figure S6) owing to the large numbers of deaths in this group (Supplementary Appendix Figure S5).

Supplementary Figure S8

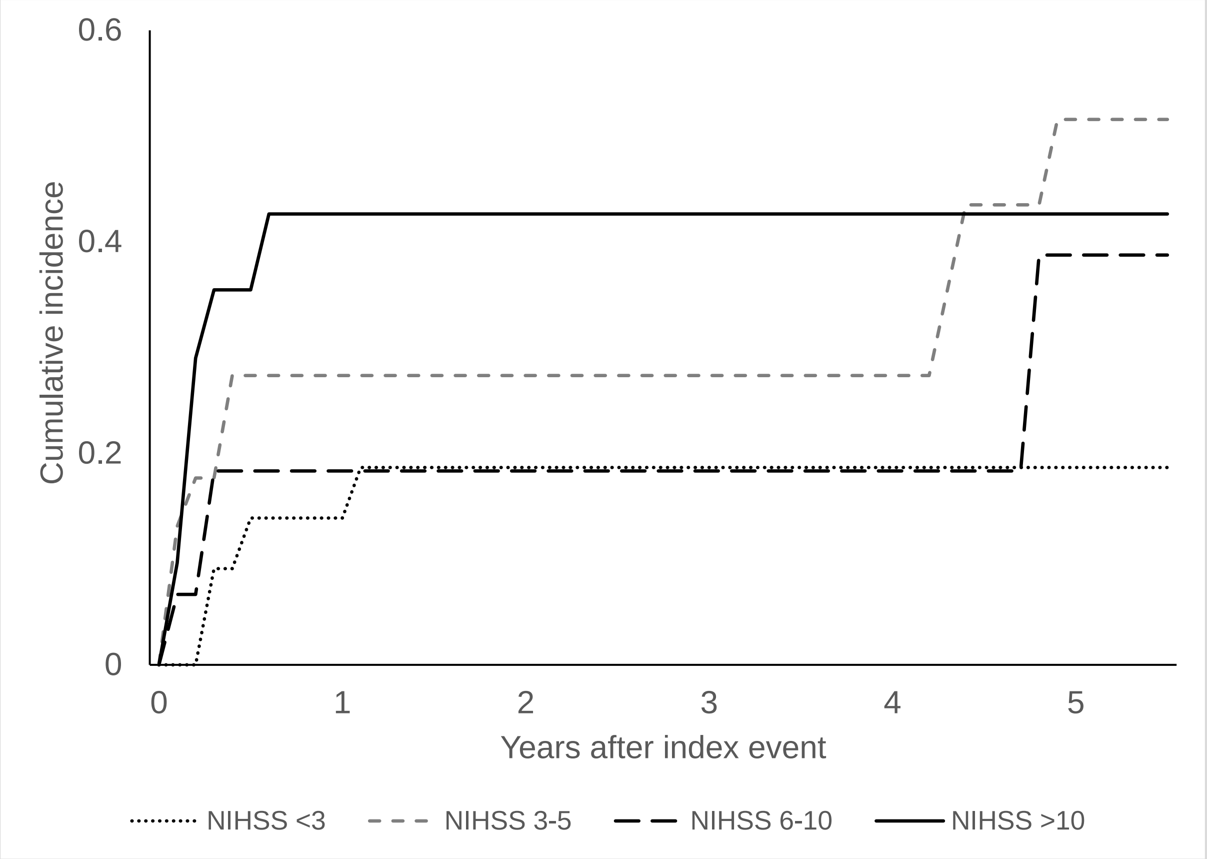

Figure S8.

Kaplan-Meier plot of time to dementia after PICH stratified by severity of event (2=NIHSS<3, 3=NIHSS 3-5, 4=NIHSS 6-10, 5=NIHSS >10.

Supplementary Figure S9.

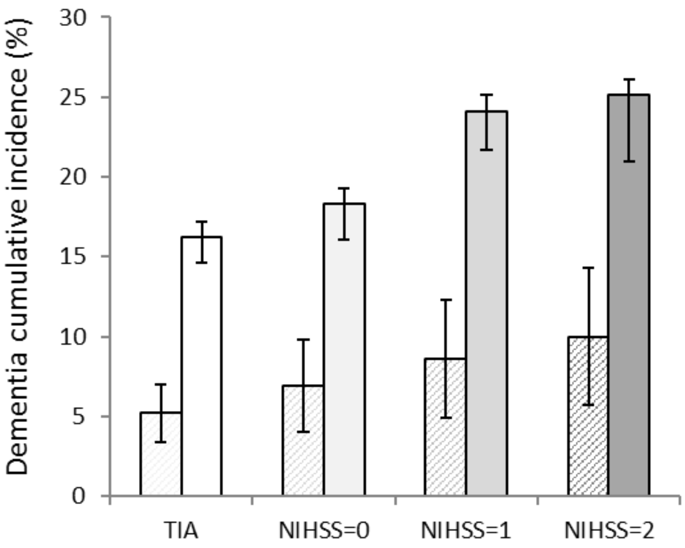

Figure S9.

One - (stippled bars) and 5-year (solid bars) cumulative incidence of dementia in TIA and minor stroke (NIHSS<3) stratified by severity of event.

Supplementary Figure S10.

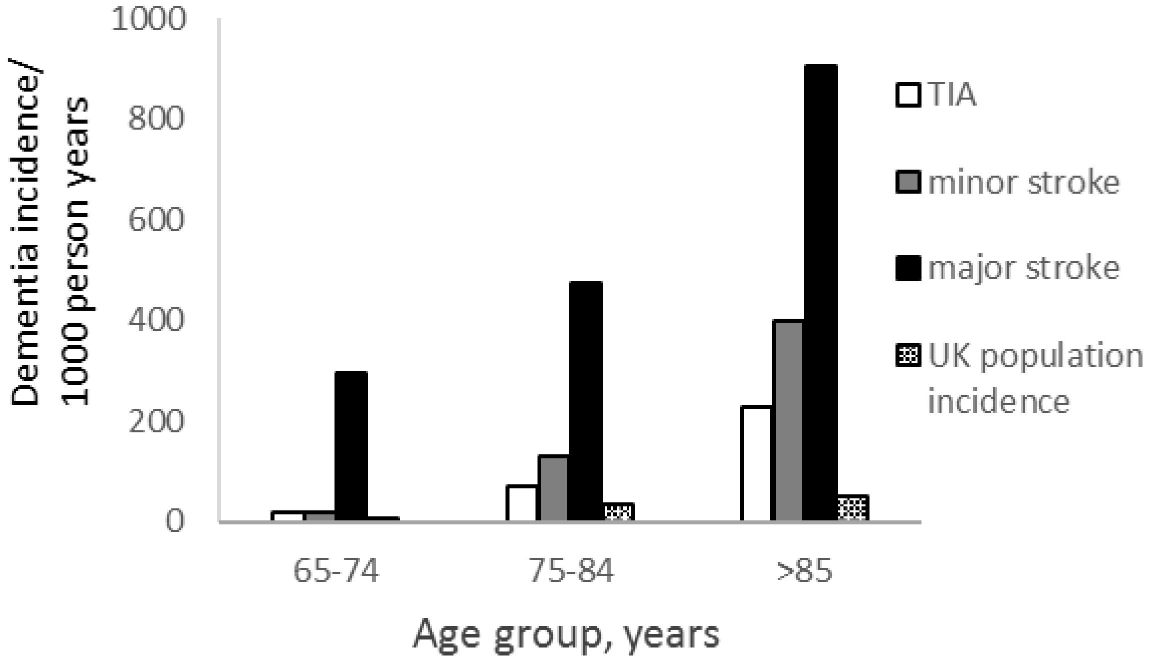

Figure S10.

One year dementia incidence/1000 person years after TIA, minor stroke and major stroke by age group versus the background population incidence rate.

Supplementary Figure S11

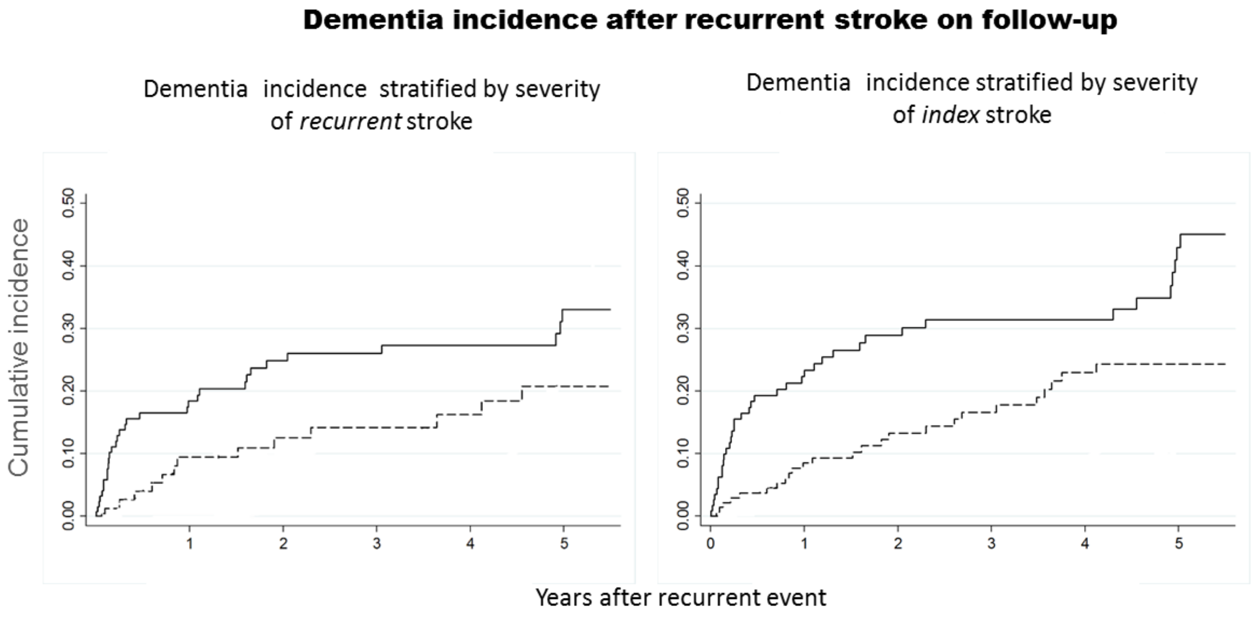

Figure S11. Kaplan-Meier (1-survival) curves of cumulative incidence of dementia by time in years after stroke on follow-up (Time 0 = time of stroke on follow-up) stratified by stroke severity (dashed line is minor stroke, NIHSS<3, solid line is major stroke (NIHSS≥3); left hand panel is stratified by severity of recurrent stroke, right hand panel is stratified by severity of the index stroke.

Supplementary Figure S12

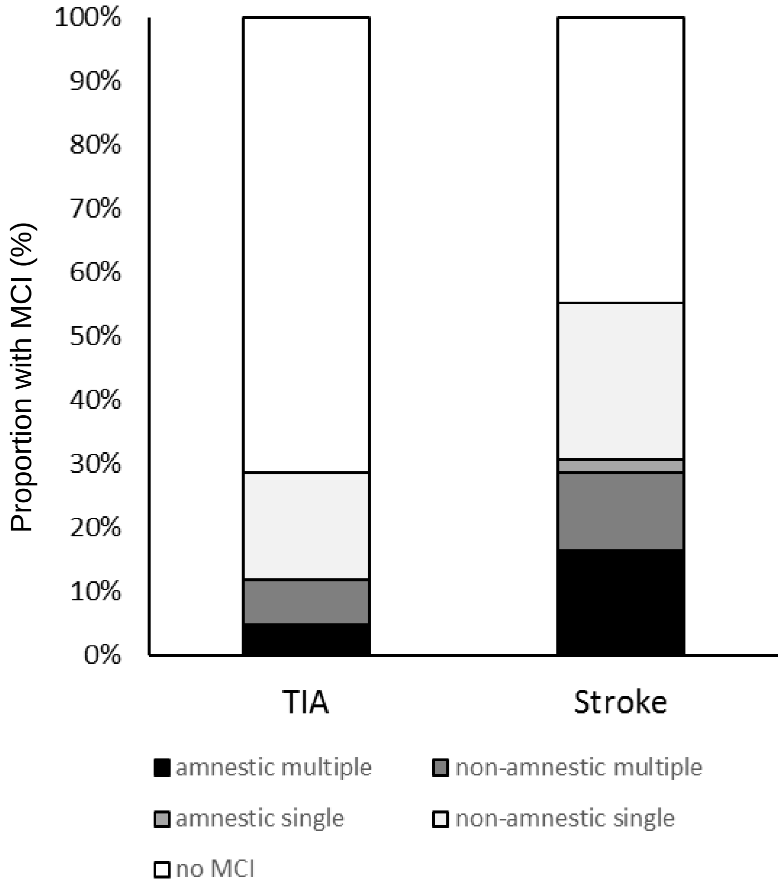

Figure S12

Percentage of patients with mild cognitive impairment (MCI) in a group of OxVASC TIA and stroke patients without dementia (n=91, mean age=70 years) who underwent neuropsychological battery at least 6 months after the index event. The modified Petersen criteria were used to define MCI and its different sub-types: amnestic and non-amnestic and single and multiple domain.

Adapted from Cerebrovasc Dis 2013;36:355–362 and Stroke 2012; 43:464-469.[13]

Table S1. Demographic and clinical characteristics and vascular risk profile for the whole cohort and separately for TIA, and minor stroke (NIHSS<3) and major stroke (NIHSS≥3).

	**Total**	**TIA**	**Minor stroke**	**Major stroke**	**p adj**
	n=2305	n=688	n=767	n=850	
**Mean (SD) age**	74.4 (13.0)	73.0 (12.9)	72.5 (13.3)	77.4 (12.3)	<0.001
**Age ≥ 75**	1281 (55.6)	344 (49.9)	375 (49.0)	562 (66.1)	<0.001
**Male sex**	1133 (49.2)	331 (48.0)	393 (51.3)	409 (48.1)	0.28
**Education<12 yrs**	1405/2095 (67.1)	423/661 (64.0)	485/728 (66.6%)	497/706 (70.4)	0.03
**Low baseline cognitive score**	322/1678 (19.2)	46/593 (7.8)	93/640 (14.5)	183/445 (41.1)	<0.001
**Premorbid Rankin ≥3**	473/2301 (20.6)	87 (12.6)	130/764 (17.0)	256/848 (30.2)	<0.001
**Premorbid Barthel <20**	476/2065 (23.1)	104/643 (16.2)	149/724 (20.6)	223/698 (31.9)	<0.001
**Vascular disease markers**					
**Prior TIA**	251 (10.9)	89 (12.9)	69 (9.0)	93 (10.9)	0.06
**Prior Stroke**	274 (11.9)	65 (9.4)	74 (9.7)	135 (15.9)	0.001
**Myocardial Infarction**	256 (11.1)	59 (8.6)	83 (10.8)	114 (13.4)	0.03
**Angina**	371 (16.1)	99 (14.4)	124 (16.2)	148 (17.4)	0.71
**Peripheral arterial disease**	178 (7.7)	40 (5.8)	59 (7.7)	79 (9.3)	0.05
**Vascular risk factors**					
**Hypertension**	1405 (60.9)	383 (55.6)	467 (61.0)	555 (65.3)	0.01
**Atrial fibrillation**	469 (20.4)	114 (16.6)	120 (15.7)	235 (27.7)	<0.001
**Diabetes**	328 (14.2)	89 (12.9)	115 (15.0)	124 (14.6)	0.27
**Hyperlipidaemia**	683 (29.6)	217 (31.5)	235 (30.7)	231 (27.2)	0.11
**Long-term smoking**	323 (14.1)	90 (13.1)	123 (16.1)	110 (13.0)	0.04

Numbers are n (%) unless otherwise specified. p adj=adjusted for age and sex

Table S2. Pre-event dementia prevalence, post-event dementia incidence (after exclusion of pre-event dementia) and point prevalence of any dementia in 1- and 5-year survivors compared to the age-and sex-matched background dementia rate for patients ≥65 years.

Prevalence, SPR; Incidence, SMR and point prevalence and SPR in survivors at 1 and 5-years (patients aged ≥65 years only)

Pre-event dementia prevalence

	Prevalence (95% CI)	SPR (95% CI)
**All**	12.0 (10.5 – 13.7)	1.2 (1.1, 1.4)
**TIA**	6.1 (4.2 – 8.7)	0.7 (0.5, 1.0)
**Stroke**	14.4 (12.4 - 16.6)	1.4 (1.2, 1.6)
**NIHSS <3**	9.5 (7.3-12.1)	1.1 (0.8-1.4)
**NIHSS 3-5**	15.4 (10.1-22.5)	1.4 (1.0-1.9)
**NIHSS 6-10**	19.2 (13.4-26.5)	1.6 (1.1-2.2)
**NIHSS >10**	22.6 (17.2-29.0)	1.9 (1.5-2.5)

Post-event dementia cumulative incidence

	1 year			1-5 year		
	**Observed**	**Expected**	**SMR (95% CI)**	**Observed**	**Expected**	**SMR (95% CI)**
**All**	237	30.4	7.8 (6.8-8.9)	171	62.0	2.8 (2.4-3.2)
**TIA**	36	10.3	3.5 (2.5-4.8)	49	32.0	1.5 (1.1-2.0)
**Stroke**	201	20.1	10.0 (8.7-11.5)	122	45.6	2.7 (2.2-3.2)
**NIHSS <3**	57	9.9	5.8 (4.4-7.5)	62	28.0	2.2 (1.7-2.8)
**NIHSS 3-5**	42	4.62	9.1 (6.6-12.3)	37	13.7	2.7 (1.9-3.7)
**NIHSS 6-10**	44	1.72	25.7 (18.6-34.4)	16	2.9	5.5 (3.1-8.9)
**NIHSS >10**	58	1.23	47.3 (35.9-61.2)	7	1.1	6.5 (2.6-13.4)

Dementia point prevalence in survivors at 1 and 5 years post-event

	1-year Prevalence	SPR (95% CI)	5-year Prevalence	SPR (95% CI)
**TIA**	10.1 (7.5-13.4)	1.2 (0.9-1.6)	14.3 (10.4-19.3)	1.4 (1.0-1.9)
**Stroke**	22.1 (19.1-25.5)	2.7 (2.3-3.1)	31.0 (26.3-36.1)	3.2 (2.7-3.7)
**NIHSS <3**	12.0 (9.1-15.6)	1.5 (1.1-2.0)	19.8 (15.1-25.5)	2.1 (1.6-2.7)
**NIHSS 3-5**	21.4 (15.5-28.7)	2.4 (1.8-3.2)	41.0 (30.4 -54.0)	4.0 (3.0-5.2)
**NIHSS 6-10**	38.2 (26.5-53.4)	4.0 (2.8-5.6)	46.7 (31.0-67.5)	4.4 (2.9-6.4)
**NIHSS >10**	64.0 (48.2-83.4)	8.0 (6.0-10.4)	62.2 (39.4-93.3)	6.5 (4.1-9.8)

SPR=standardised prevalence ratio, SMR=standardised morbidity ratio.

Table S3. Pre-event dementia prevalence, post-dementia incidence and point prevalence of dementia in survivors at 1 and 5 years compared to the age-matched background dementia rate for patients $\geq$65 years excluding prior stroke.

Prevalence, SPR; Incidence, SMR and point prevalence and SPR in survivors

Pre-event dementia prevalence

	Prevalence (95% CI)	SPR (95% CI)
**TIA**	4.5 (2.8-6.9)	0.5 (0.3-0.8)
**Stroke**	12.5 (10.5-14.7)	1.3 (1.1-1.5)
**NIHSS <3**	7.5 (5.3-10.2)	0.9 (0.6-1.2)
**NIHSS 3-5**	12.9 (8.7-18.5)	1.3 (0.9-1.9)
**NIHSS 6-10**	17.2 (11.3-25.0)	1.4 (0.9-2.1)
**NIHSS >10**	20.0 (14.5-26.9)	1.7 (1.3-2.3)

Incident dementia post-event

	1 year			1-5 year		
	**Observed**	**Expected**	**SMR**	**Observed**	**Expected**	**SMR (95% CI)**
**TIA**	28	8.1	3.4 (2.3, 5.0)	41	24.7	1.7 (1.2, 2.3)
**Stroke**	132	14.5	9.1 (7.6, 10.8)	75	40.6	1.9 (1.5 (2.3)
**NIHSS <3**	38	7.2	5.3 (3.8, 7.3)	37	20.2	1.8 (1.3, 2.5)
**NIHSS 3-5**	32	3.3	9.9 (6.7, 13.9)	27	7.7	3.5 (2.3, 5.1)
**NIHSS 6-10**	62	4.0	15.4 (11.8, 19.8)	11	12.7	0.9 (0.4, 1.6)
**NIHSS >10**	24	1.6	15.2 (9.7, 22.6)	9	4.0	2.3 (1.0, 4.3)

Dementia point prevalence in survivors at 1 and 5 years post-event

	1-year Prevalence	SPR (95% CI)	5-year Prevalence	SPR (95% CI)
**TIA**	8.4 (5.8 - 11.8)	1.1 (0.73, 1.48)	13.8 (9.7 – 19.0)	1.4 (1.0, 1.9)
**Stroke**	20.7 (17.3 - 24.6)	2.5 (2.07, 2.94)	27.2 (22.2 – 33.0)	2.8 (2.3, 3.4)
**NIHSS < 3**	11.6 (8.4 - 15.7)	1.5 (1.1, 2.0)	18.4 (13.3 - 24.9)	2.0 (1.4, 2.6)
**NIHSS 3-5**	21.4 (14.5 - 30.6)	2.4 (1.6, 3.5)	37.4 (25.4 - 53.0)	3.8 (2.6, 5.4)
**NIHSS 6-10**	36.1 (22.6 - 54.6)	3.5 (2.2, 5.3)	38.1 (21.8 - 61.9)	3.6 (2.1, 5.9)
**NIHSS >10**	60.7 (42.1 - 84.8)	7.7 (5.3, 10.7)	53.9 (29.4 - 90.3)	5.8 (3.2, 9.8)

SPR=standardised prevalence ratio, SMR=standardised morbidity ratio.

Table S4. Factors associated with pre-event dementia, showing raw numbers and unadjusted odds ratios (ORs).

	Dementia yes (255) vs no (2080)	OR (95% CI)	p
Susceptibility Factors			
Age per year		1.09 (1.07, 1.11)	<0.0001
Age ≥75	195/1281 (15.2%) vs 30/1024 (2.9%)	5.95 (4.01, 8.82)	<0.0001
Male sex	88/1133 (7.8%) vs 137/1172 (11.7%)	0.64 (0.48, 0.84)	0.002
Low education	128/1405 (9.1%) vs 46/690 (6.7%)	1.40 (0.99, 1.99)	0.058
Rankin ≥3	165/473 (34.9%) vs 60/1828 (3.3%)	15.79 (11.47, 21.72)	<0.0001
Barthel <20	123/476 (25.8%) vs 42/1589 (2.6%)	12.83 (8.88, 18.56)	<0.0001
Diabetes	46/328 (14.0%) vs 179/1977 (9.1%)	1.64 (1.16, 2.32)	0.005
Long-term smoking	19/323 (5.9%) vs 203/1972 (10.3%)	0.54 (0.34, 0.89)	0.014
Leukariosis			
none/mild	89/1438 (6.2%)	1	
moderate severe	96/699 (13.7%)	2.41 (1.78, 3.27)	<0.0001
Current vascular risk profile			
Hypertension	137/1405 (9.8%) vs 88/900 (9.8%)	1.00 (0.75, 1.32)	0.98
Atrial Fibrillation	69/469 (14.7%) vs 156/1836 (8.5%)	1.86 (1.37, 2.52)	<0.001
Myocardial Infarction	36/256 (14.1%) vs 189/2049 (9.2%)	1.61 (1.11, 2.36)	0.02
Angina	56/371 (15.1%) vs 169/1934 (8.7%)	1.86 (1.34, 2.57)	<0.001
Peripheral vasc. disease	23/178 (12.9%) vs 202/2127 (9.5%)	1.41 (0.89, 2.24)	0.14
Hyperlipidaemia	53/683 (7.8 %) vs 172/1622 (10.6%)	0.71 (0.51, 0.98)	0.04
Measured baseline cognition			
Low cognitive score	73/322 (22.7%) vs 15/1356 (1.1%)	26.21 (14.79, 46.43)	<0.001
Stroke lesion burden/location			
Prior TIA	30/251 (12.0%) vs 195/2054 (9.5%)	1.29 (0.86, 1.95)	0.22
Prior stroke	61/274 (22.3%) vs 164/2031 (8.1%)	3.26 (2.35, 4.52)	<0.001
TIA	34/689 (4.9%)	1	<0.001
Ischaemic stroke	182/1482 (12.3%)	2.69 (1.85, 3.93)	
PICH	9/135 (6.7%)	1.37 (0.64, 2.94)	
NIHSS, per point incr.	7.09 (7.65) vs 3.51 (6.01)	1.07 (1.05, 1.09)	<0.001
NIHSS <3	52/766 (6.8%)	1.40 (0.90, 2.19)	<0.001
NIHSS 3-5	38/333 (11.4%)	2.48 (1.53, 4.02)	
NIHSS 6-10	40/221 (18.1%)	4.26 (2.62, 6.92)	
NIHSS >10	61/296 (20.6%)	5.00 (3.20, 7.80)	
Dysphasia	65/402 (16.2%) vs 158/1891 (8.4%)	2.12 (1.55, 2.89)	<0.001
Number of vascular risk factors			
0	48/474 (10.1%)	1	0.50
1	71/832 (8.5%)	0.83 (0.56, 1.22)	
2	71/675 (10.5%)	1.04 (0.71, 1.54)	
≥ 3	35/324 (10.8%)	1.07 (0.68, 1.70)	

vasc. = vascular; PICH=primary intracerebral haemorrhage

Table S5. Factors associated with post-event dementia showing raw numbers and unadjusted hazard ratios (HR)

	**Dementia yes (432) vs no (1648)**	**HR (95% CI)**	**p**
Susceptibility factors			
Age, per year		1.09 (1.08-1.10)	<0.001
Age ≥75	340/1086 (31.3%) vs 92/994 (9.3%)		
Male sex	170/1045 (16.3%) vs 262/1035 (25.3%)	0.60 (0.49-0.72)	<0.001
Low education	318/1277 (24.9%) vs 89/644 (13.8%)	1.88 (1.50-2.36)	<0.001
Rankin ≥3	112/308 (36.4%) vs 320/1768 (18.1%)	3.42 (2.75-4.25)	<0.001
Barthel < 20	130/353 (36.8%) vs 275/1547 (17.8%)	3.00 (2.40-3.70)	<0.001
Diabetes	70/282 (24.8%) vs 362/1798 (20.1%)	1.33 (1.03-1.72)	0.03
Life-long smoking	43/304 (14.1%) vs 388/1769 (21.9%)	0.58 (0.42-0.79)	0.001
Leukariosis			
none/mild	232/1349 (17.2%)	1	
moderate severe	187/603 (31.0%)	2.25 (1.85-2.72)	<0.001
Current vascular risk profile			
Hypertension	296/1268 (23.3%) vs 136/812 (16.8%)	1.55 (1.27-1.90)	<0.001
Atrial fibrillation	101/400 (25.3%) vs 331/1680 (19.7%)	1.65 (1.32-2.06)	<0.001
Myocardial infarction	56/220 (25.5%) vs 376/1860 (20.2%)	1.41 (1.07-1.87)	0.02
Angina	73/315 (23.2%) vs 359/1765 (20.3%)	1.20 (0.93-1.55)	0.15
Peripheral vasc. disease	36/155 (23.2%) vs 396/1925 (20.6%)	1.39 (0.98-1.95)	0.06
Hyperlipidaemia	134/630 (21.3%) vs 298/1450 (20.6%)	0.93 (0.76-1.14)	0.47
Measured baseline cognition			
Low cognitive score	123/249 (49.4%) vs 196/1341 (14.6%)	5.57 (4.48-6.94)	<0.001
Stroke lesion burden/location			
Prior TIA	61/221 (27.6%) vs 371/1859 (20.0%)	1.41 (1.08-1.85)	0.01
Prior stroke	70/213 (32.9%) vs 362/1867 (19.4%)	1.92 (1.49-2.49)	<0.001
TIA	85/655 (13.0%)	1	<0.001
Ischaemic stroke	325/1300 (25.0%)	2.46 (1.93-3.21)	
PICH	23/126 (18.3%)	3.15 (1.98-5.00)	
NIHSS, per point	4.83 (5.93) vs 3.16 (5.98)	1.12 (1.10-1.14)	<0.001
NIHSS <3	124/714 (17.4%)	1.41 (1.07-1.85)	
NIHSS 3-5	88/295 (29.8%)	2.86 (2.12-3.85)	
NIHSS 6-10	65/181 (35.9%)	5.40 (3.91-7.47)	
NIHSS >10	70/235 (29.8%)	7.58 (5.51-10.43)	
Dysphasia	117/337 (34.7%) vs 315/1733 (18.2%)	3.73 (3.02-4.62)	<0.001

**Vascular risk profile factors**			0.015
**0**	72/426 (16.9%)	1	
**1**	153/761 (20.1%)	1.22 (0.92-1.61)	
**2**	141/604 (23.3%)	1.54 (1.16-2.05)	
**≥ 3**	66/289 (22.8%)	1.44 (1.03-2.02	
**Vascular risk factors (BP, hyperlipidaemia, diabetes, long-term smoking)**		0.03	
**0**		1	
**1**		1.02 (0.,79-1.31)	
**≥2**		1.32 (1.02-1.71)	
Brain imaging (CT/MRI)			
Acute lesion	184/736 (25%) vs 189/995 (19%)	1.58 (1.29-1.94)	
Old lesion	76/324 (23%) vs 296/1406 (21%)	1.17 (0.91-1.50)	
Multiple lesions	23/82 (28%) vs 350/1649 (21%)	1.70 (1.11-2.59)	

P values are unadjusted, vasc.=vascular, PICH=primary intracerebral haemorrhage

Table S6. Hazard ratios (95% CI) from sub-distribution vs cause-specific hazard models for post-event dementia adjusted for age, sex and education.

		Post-event Dementia	
		Subdistribution HR (95% CI)	Cause-specific HR (95% CI)
**Susceptibility factors**			
Age per year		1.06 (1.05-1.07)	1.09 (1.07-1.10)
Age ≥ 75		3.70 (2.93-4.67)	4.69 (3.71-5.92)
Male sex		0.84 (0.69-1.02)	0.85 (0.70-1.03)
Low education		1.62 (1.29-2.04)	1.69 (1.36-2.12)
Pre-morbid Rankin ≥ 3		1.41 (1.10-1.80)	1.81 (1.43-2.28)
Pre-morbid Barthel < 20		1.34 (1.05-1.69)	1.60 (1.28-2.00)
Leukoariosis (mod/severe)		1.44 (1.18-1.76)	1.45 (1.19-1.77)
**Current vascular risk profile**			
Treated hypertension		1.08 (0.88-1.33)	1.11 (0.91-1.37)
Diabetes		1.45 (1.11-1.90)	1.54 (1.19-2.00)
Treated hyperlipidaemia		1.07 (0.87-1.32)	1.04 (0.84-1.27)
Atrial fibrillation		0.96 (0.75-1.21)	1.24 (0.99-1.55)
Myocardial infarction		1.10 (0.83-1.45)	1.17 (0.88-1.55)
Angina		0.85 (0.66-1.11)	0.91 (0.71-1.18)
Peripheral arterial disease		1.01 (0.71-1.43)	1.26 (0.90-1.78)
Long-term smoking		1.13 (0.82-1.55)	1.26 (0.90-1.74)
**Low baseline cognitive score**		3.39 (2.68-4.29)	4.34 (3.48-5.42)
**Stroke lesion burden/location**			
Prior TIA		1.09 (0.83-1.43)	1.03 (0.79-1.36)
Prior stroke		1.35 (1.04-1.76)	1.44 (1.11-1.86)
TIA		1.00	1.00
Ischaemic stroke		1.98 (1.56-2.51)	2.53 (1.99-322)
PICH		2.23 (1.38-3.61)	4.51 (2.84-7.17)
TIA		1.00	1.00
Stroke severity	NIHSS < 3	1.45 (.1.11-1.89)	1.57 (1.19-2.07)
	NIHSS 3-5	2.37 (1.76-3.19)	2.72 (2.01-3.67)
	NIHSS 6-10	3.27 (2.33-4.59)	4.88 (3.53-6.75)
	NIHSS > 10	2.18 (1.53-3.11)	7.67 (5.55-10.59)
NIHSS, per point		1.03 (1.01-1.04)	1.12 (1.10-1.13)
Dysphasia		1.87 (1.47-2.38)	3.26 (2.63-4.04)
**Brain imaging (CT/MRI)**			
Acute lesion		1.43 (1.17-1.76)	1.84 (1.50-2.26)
Old lesion		1.08 (0.83-1.39)	1.09 (0.85-1.41)
Multiple lesions		1.74 (1.10-2.74)	1.91 (1.11-3.28)

Supplementary Table S7. Baseline characteristics in patients with PICH associated with post-event dementia (after exclusion of pre-event dementia).

	**Post-event dementia N=23/126**	
	HR (95% CI)*	P*
Susceptibility Factors		
**Age per year**	1.07 (1.02-1.11)	0.002
**Age ≥ 75**		
**Male sex**	0.70 (0.28-1.57)	0.35
**Low education**	1.92 (0.74-4.47)	0.19
**Pre-morbid Rankin ≥ 3**	1.68 (0.52-5.38)	0.39
**Pre-morbid Barthel < 20**	1.64 (0.41-6.52)	0.48
**Leukoariosis (mod/severe)**	1.89 (0.70-5.08)	0.21
Vascular risk factors		
Hypertension	1.82 (0.70-4.72)	0.22
Diabetes	0.86 (0.20-3.75)	0.85
Treated Hyperlipidaemia	0.83 (0.32-2.17)	0.71
Total cholesterol		
Atrial fibrillation	1.48 (0.50-4.41)	0.48
Peripheral arterial disease	1.59 (0.43-5.92)	0.49
Current smoking	1.00 (0.22-4.69)	0.99
Low baseline cognitive score †	3.36 (1.02-11.10)	0.05
Lesion burden/location		
**Prior TIA**	1.52 (0.44-5.22)	0.51
**Prior stroke**	1.40 (0.31-6.32)	0.66
**(NIHSS, per point)**	1.08 (1.01-1.15)	0.03
**Dysphasia**	3.33 (1.35-8.22)	0.009

Low education=education ≤12 years, Leukoariosis (mod/severe)=Leukoariosis (moderate/severe)
*adjusted for age, sex and education. †Mini-mental state examination<24, or acute confusion, if untestable.

References

1. Rothwell PM, Coull AJ, Giles MF, Howard SC, Silver LE, Bull LM, et al; Oxford Vascular Study. Change in stroke incidence, mortality, case-fatality, severity, and risk factors in Oxfordshire, UK from 1981 to 2004 (Oxford Vascular Study). Lancet. 2004;363:1925-33.

2. Rothwell PM, Coull AJ, Silver LE, Fairhead JF, Giles MF, Lovelock CE, et al; Oxford Vascular Study. Population-based study of event-rate, incidence, case fatality, and mortality for all acute vascular events in all arterial territories (Oxford Vascular Study). Lancet 2005;366:1773-83.

3. Coull AJ, Silver LE, Bull LM, Giles MF, Rothwell PM; Oxford Vascular (OXVASC) Study. Direct assessment of completeness of ascertainment in a stroke incidence study. Stroke. 2004;35:2041-5.

4. Bamford J, Sandercock P, Dennis M, Burn J, Warlow C. A prospective study of acute cerebrovascular disease in the community: the Oxfordshire Community Stroke Project--1981-86. 2. Incidence, case fatality rates and overall outcome at one year of cerebral infarction, primary intracerebral and subarachnoid haemorrhage. *J Neurol Neurosurg Psychiatry* 1990;53:16-22.

5. Pendlebury ST, Mariz J, Bull L, Mehta Z, Rothwell PM. MoCA, ACE-R and MMSE versus the national institute of neurological disorders and stroke-canadian stroke network vascular cognitive impairment harmonization standards neuropsychological battery after TIA and stroke. Stroke. 2012;43:464-469.

6. Pendlebury ST, Welch SJ, Cuthbertson FC, Mariz J, Mehta Z, Rothwell PM. Telephone assessment of cognition after TIA and stroke: TICSm and telephone MoCA vs face-to-face MoCA and neuropsychological battery. Stroke 2013;44:227-9.

7. Easton JD, Saver JL, Albers GW, et al. Definition and evaluation of transient ischemic attack: a scientific statement for healthcare professionals from the American Heart Association/American Stroke Association Stroke Council; Council on Cardiovascular Surgery and Anesthesia; Council on Cardiovascular Radiology and Intervention; Council on Cardiovascular Nursing; and the Interdisciplinary Council on Peripheral Vascular Disease. The American Academy of Neurology affirms the value of this statement as an educational tool for neurologists. *Stroke* 2009;40:2276-2293.

8. Sacco RL, Kasner SE, Broderick JP, et al. An updated definition of stroke for the 21st century: a statement for healthcare professionals from the American Heart Association/American Stroke Association. *Stroke* 2013;44:2064-2089.

9. Hatano S. Experience from a multicentre stroke register: a preliminary report. *Bulletin of the World Health Organization* 1976;54:541-553.

10. Pendlebury ST, Chen PJ, Bull L, Silver L, Mehta Z, Rothwell PM; Oxford Vascular Study. Methodological factors in determining rates of dementia in transient ischemic attack and stroke: (I) impact of baseline selection bias. Stroke. 2015;46:641-6.

11. Pendlebury ST, Chen PJ, Welch SJ, et al; Oxford Vascular Study. Methodological Factors in Determining Risk of Dementia After Transient Ischemic Attack and Stroke: (II) Effect of Attrition on Follow-Up. Stroke. 2015;46:1494-500.

12. Pendlebury ST, Klaus SP, Thomson RJ, Mehta Z, Wharton RM, Rothwell PM. Methodological Factors in Determining Risk of Dementia After Transient Ischemic Attack and Stroke: (III) Applicability of Cognitive Tests. Stroke. 2015 ;46:3067-73.

13. Pendlebury ST, Mariz J, Bull L, Mehta Z, Rothwell PM. Impact of different operational definitions on mild cognitive impairment rate and MMSE and MoCA performance in transient ischaemic attack and stroke. Cerebrovasc Dis.2013;36:355-62.

14. Fine, J. and Gray R.. A proportional hazards model for the subdistribution of a competing risk. Journal of the American Statistical Association. 1999;**94**:496–509).

15. Lau B, Cole SR, Gange SJ. Competing risk regression models for epidemiologic data. Am J Epidemiol. 2009;170:244-56.
